# Unravelling and overcoming the challenges in the electrocatalytic reduction of fructose to sorbitol†

**DOI:** 10.1039/d2gc04451j

**Published:** 2023-01-12

**Authors:** Jordi Creus, Matteo Miola, Paolo P. Pescarmona

**Affiliations:** a Chemical Engineering Group, Engineering and Technology Institute Groningen (ENTEG), University of Groningen Nijenborgh 4 9747 AG Groningen The Netherlands p.p.pescarmona@rug.nl

## Abstract

In this work, we present a comprehensive study of the electrocatalytic reduction of fructose to sorbitol and mannitol, in a mild alkaline medium (pH = 11.3), with a Cu wire as the cathode. Particular attention was paid to the reaction mechanism, investigated by linear sweep voltammetry (LSV) and chronopotentiometry (CP) coupled with high-pressure liquid chromatography (HPLC). The initial results of our study showed that at the potential where the fructose reduction reaction (FRR) is achieved, competition with the hydrogen evolution reaction (HER) tends to occur, thus limiting the Faradaic efficiency towards the FRR. Moreover, products of chemical conversions were also observed in the liquid electrolyte, originating from the isomerisation of fructose to glucose and mannose and degradation reactions (C–C breaking). Through a thorough optimisation of the reaction parameters, the Faradaic efficiency could be remarkably improved, reaching values >40% and being sustained for 10 h of electrolysis at a current of *i* = −20 mA. More specifically, the minimisation of the undesired chemical side reactions was achieved by the careful control of the pH (11.3 ± 0.3) using a buffer electrolyte and a titration pump, thus limiting the isomerisation of fructose to glucose and mannose to <2% in 10 h. The electrochemical conversion was optimised *via* a tailored strategy involving a two-step potential cycling for re-activating the electrocatalyst surface, which allowed achieving 77% electrochemical conversion of fructose to sorbitol and mannitol in 10 h of electrolysis (sorbitol : mannitol = 0.43 : 0.57). This is the first time that the electrocatalytic FRR was achieved with such a high product yield and by using a non-noble metal-based cathode, thus opening up a novel, green route for the conversion of fructose into sorbitol and mannitol. This work also provides relevant, new insight into the crucial parameters that need to be taken into account to achieve the electrocatalytic reduction of saccharides, by gaining control of their complex chemistry in solution.

## Abstract 2

In this work, we present a comprehensive study and optimisation of the electrocatalytic reduction of fructose to sorbitol and mannitol over a Cu-wire cathode, which allowed enhancing the Faradaic efficiency and achieving unprecedented product yields.

## Graphical abstract

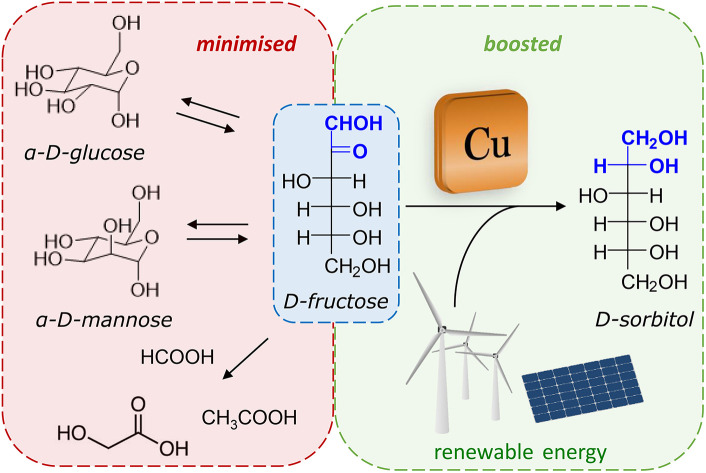

## Introduction

**Scheme 1:**
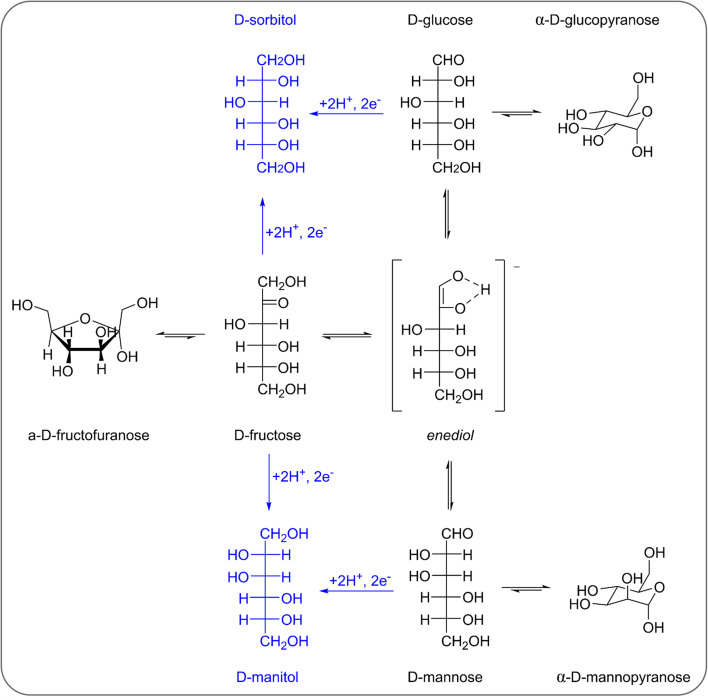
Reaction network involved in the electrochemical reduction of fructose, glucose and mannose to sorbitol and mannitol (including the mutarotation and isomerisation of fructose, glucose and mannose). The electrochemical steps are in blue, the chemical ones are in black.

The conversion of organic molecules *via* thermocatalysis typically requires relatively high temperatures (*T* > 200 °C), usually attained through the consumption of fossil fuels,^1–3^ and often requires the use of chemical reagents that can lead to hazardous waste.^4,5^ Recently, the use of electrocatalytic approaches as an alternative to realise these transformations has been gathering increasing attention in the research community.^6–8^ Electrocatalytic processes present several advantages over conventional chemical production routes: (1) they are generally carried out at ambient temperatures and pressures, resulting in mild operation conditions; (2) they use H_2_O (H^+^ and OH^−^) as a proton or oxygen source for the reduction/oxidation reactions, avoiding the use of reducing/oxidising agents; and (3) the reaction conditions can be easily tuned (*e.g.* by changing the electric potential), enabling the control of the reaction rate and selectivity.^9,10^ In the context of sustainability, an important advantage of these processes is the possibility of using renewable power sources (*e.g.* solar, wind, hydro, tidal) to achieve thermodynamically unfavourable reactions. The greenness of electrocatalytic synthesis for the preparation of value-added organic molecules^11^ can be further enhanced if renewable, biobased and abundant chemicals such as saccharides or sugar alcohols are used as feedstock.^12,13^ While the electrochemical oxidation of saccharides (especially glucose) has already gathered substantial attention within the scientific community,^3,14–16^ the reduction of these species is much more challenging and only a handful of studies on this topic have reached scientific publication (see Fig. S1†).^6^ A major challenge in the electrocatalytic reduction of saccharides is the competing hydrogen evolution reaction (HER),^11,17^ which occurs in the same potential range as a result of a common first reaction step, the so-called Volmer step (formation of a M–H species at the electrode surface upon chemisorption of a proton/water in an acidic/alkaline medium, respectively).^18,19^ Among the possible products of the electrocatalytic reduction reaction of the two main monosaccharides, glucose and fructose (glucose/fructose reduction reaction: GRR/FRR, see Scheme 1),^6,20^ sorbitol is highly attractive for its application as an additive in the food, pharma and cosmetic industries and is therefore recognised as one of the top 10 biobased platform molecules.^12,13^ In the electrochemical reduction of fructose, mannitol is observed as a side-product besides sorbitol. Mannitol is also a valuable compound that is widely applied as an additive in food and pharmaceuticals.^21^ Before 1950, the production of sorbitol from glucose, as other organic reactions, was carried out industrially *via* an electrochemical process involving the use of highly toxic mercury or lead cathodes in diluted sulphuric acid/sodium sulphate mixtures,^22^ as these electrodes display high HER overpotential and thus limit the aforementioned competition with the HER.^11,17^ Besides the environmental drawbacks, the low technological maturity and high costs of this electrochemical route as well as the increase in the electricity price induced a switch to the thermocatalytic hydrogenation of glucose over RANEY®-Ni.^23,24^ However, this process typically requires temperatures >130 °C and H_2_ pressures of 4–12 MPa.^25^ Additionally, scarce and costly Ru is generally used to overcome the leaching of Ni from the catalyst.^21,25^ Against this backdrop, research on electrocatalytic production of sorbitol slowly evolved and in 1985 the use of RANEY®-Ni as the cathode in substitution of Zn(Hg) was reported, achieving an improved performance under (nearly) neutral conditions (0.4 M CaBr_2_, pH = 5–7, 60 °C, *i* = −200 mA at −0.54 V *vs.* RHE).^17,26^ RANEY®-Ni was reported to show >99% Faradaic efficiency (FE) towards the formation of sorbitol from glucose in a flow-cell reactor, in contrast to a maximum of 60% obtained with Zn(Hg) in the same reactor.^26^ However, other authors reported that a conventional Ni electrode was nearly inactive under neutral conditions.^27,28^ Since then, other electrocatalysts were reported to be active in the GRR, although with lower Faradaic efficiency. For instance, FE = 90% was achieved with an alloy electrode (La_0.68_Ce_0.17_Pr_0.05_Nd_0.10_)Ni_3.55_Co_0.75_Mn_0.40_Al_0.30_ ^29^ at 8 mA cm^−2^, pH = 8 and 30 °C, whereas Zn and alloyed Zn supported on carbon nanotubes allowed reaching FE = 58% (pH = 11, 30 °C).^30^ More recently, a fundamental study on the use of different solid metal electrodes for the reduction of glucose was performed at neutral pH (0.1 M Na_2_SO_4_) and the products were detected by online HPLC, which *in situ* acquired the samples at very short reaction times during a linear sweep voltammetry (LSV) experiment.^6^ The tested metals were divided into three groups depending on the products that were formed: (a) metals over which sorbitol and H_2_ formed (Fe, Co, Ni, Cu, Pd, Au, Ag, and Al); (b) metals over which sorbitol, 2-deoxysorbitol and very little H_2_ formed (Zn, Cd, In, Sn, Sb, Pb, and Bi); and (c) metals over which only H_2_ formed (Ti, V, Cr, Mn, Zr, Nb, Mo, Hf, Ta, W, Re, Ru, Rh, Ir, and Pt). All the publications discussed above focussed on the reduction of glucose, whereas the electrochemical reduction of fructose to sorbitol has been reported only once.^20^ In that publication, a Pt–Rh/C electrode was employed as the cathode in a flow cell, achieving a 60% steady fructose conversion with 40% product selectivity towards sorbitol at −1.25 V *vs.* SCE (−0.42 V *vs.* RHE) over a period of 16 h. However, no data were provided about the Faradaic efficiency and the non-electrochemical isomerisation of fructose to glucose was observed (5% after 4 h), meaning that the electrochemical conversion was lower than the overall 60% fructose conversion.

In this work, we provide for the first time a thorough investigation of the fructose reduction reaction (FRR) in alkaline media. By controlling and tuning the reaction parameters, we were able to improve the reaction rate while minimising the formation of side products (isomerisation and degradation) and the competing HER, thus maximising the Faradaic efficiency. The obtained results represent a relevant green advance in the context of electrification of the chemical industry as they demonstrate the feasibility of the electrocatalytic production of sorbitol and mannitol with relevant, unmatched yields through the reduction of a biobased compound such as fructose over an inexpensive and non-toxic Cu electrode. Additionally, this work lays the foundation for future studies of electrocatalytic systems for the conversion of saccharides, as it introduces effective strategies to tackle the challenges caused by the complex reactivity of saccharide molecules.

## Results and discussion

The first step in our investigation of the electrochemical fructose reduction reaction (FRR) to sorbitol was the choice of the material to be used as the cathode. Looking for an Earth-abundant metal and inspired by previous reports on the glucose reduction reaction (GRR),^6^ we decided to use a commercially available Cu wire as the working electrode (WE, S.A. = 3.78 cm^2^, see Fig. S2† for electrode and cell design). The redox reactions expected to take place in the electrochemical cell are:

At the working electrode (cathodic reactions):

FRR (basic medium):
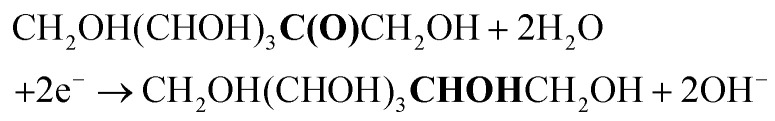


FRR (acidic medium):1
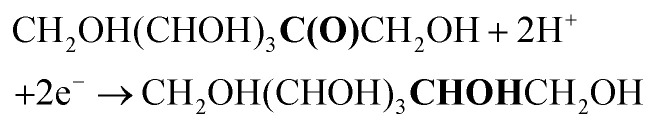


HER (basic medium):2H_2_O + 2e^−^ → H_2_ + 2OH^−^

HER (acidic medium):

2 2H^+^ + 2e^−^ → H_2_

At the counter electrode (anodic reaction):

OER (basic medium):2OH^−^ → H_2_O + O_2_ + 2e^−^

OER (acidic medium):

3 H_2_O_2_ + 2H^+^ + 2e^−^

Overall target reaction:4
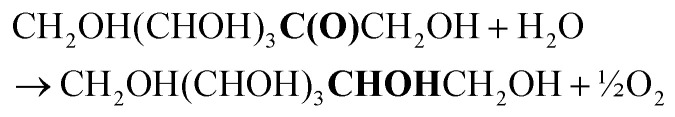
(FRR = fructose reduction reaction, HER = hydrogen evolution reaction, OER = oxygen evolution reaction. The carbonyl group in fructose and the alcohol group in sorbitol obtained by the selective reduction of the carbonyl are highlighted in bold).

### Optimisation of reaction conditions

Previous reports demonstrated that the electrochemical reduction of a monosaccharide proceeds only if the compound is in its linear form (for more details see Section S2 in the ESI†).^27,31^ The mutarotation from the cyclic form to the linear one in solution (Scheme S1† for fructose mutarotation) is sluggish, but the observed dependence of the current on the potential shows that this is not the rate determining step of the overall process.^27^ Fructose mutarotation has been studied in D_2_O, and it has been proved that the acyclic (linear) keto form of fructose can account for up to 0.5–2% of the fructose in solution.^32,33^ This equilibrium concentration is 1–2 orders of magnitude higher than that of the linear aldehyde form of glucose under similar conditions (0.024%).^31^ In the case of glucose, under neutral conditions it takes around 3 h to reach the equilibrium, while this happens much faster under acidic conditions and almost instantaneous under alkaline conditions.^34^ However, strong alkaline pH = 13 was also shown to boost the undesired isomerisation and degradation of these monosaccharides (also proved in control tests summarised in Section S3 of the ESI, Fig. S13a and b†), thus diminishing the overall reaction selectivity. Based on these considerations, we chose fructose as the feedstock and investigated the electrochemical reduction in a nearly neutral electrolyte (0.1 M Na_2_SO_4_, pH = 6.8), which is a typical medium in which the reduction of saccharides has been performed in previous studies.^6,18,30^

**Fig. 1:**
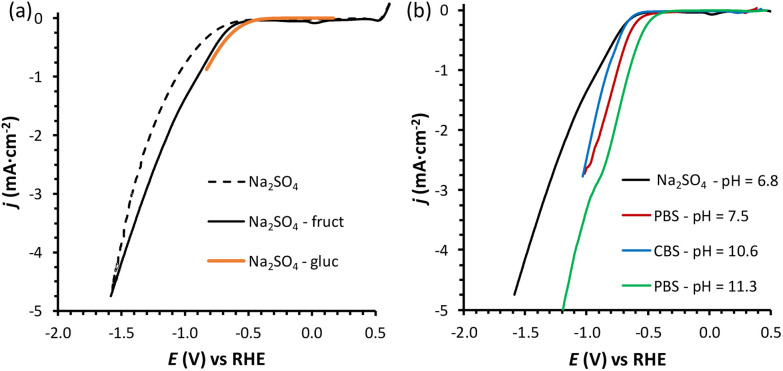
(a) LSVs of a Cu-wire electrode in 0.1 M Na_2_SO_4_ at pH = 6.8 (dotted black line), in the presence of 0.1 M fructose (full black line), or 0.1 M glucose (continuous orange line); and (b) in the presence of 0.1 M fructose at four different pHs: 0.1 M Na_2_SO_4_ at pH = 6.8 (black), 0.18 M phosphate buffer solution (PBS) at pH = 7.5 (red), 0.12 M PBS at pH = 11.3 (green), and 0.5 M carbonate buffer solution (CBS) at pH = 10.6 (blue). The voltammetries were run at a 10 mV s^−1^ scan rate at room temperature (21 °C) with a Nafion membrane separator. The potential range used in each experiment depends on the pH, with the reductive potential varied until *ca.* −5 mA cm^−2^ was reached. The small reduction peak observed at *ca.* 0 V *vs.* RHE for the fructose LSV is attributed to the reduction of oxidised Cu species, as discussed in Section S4 and Fig. S16–S19.†

**Fig. 2:**
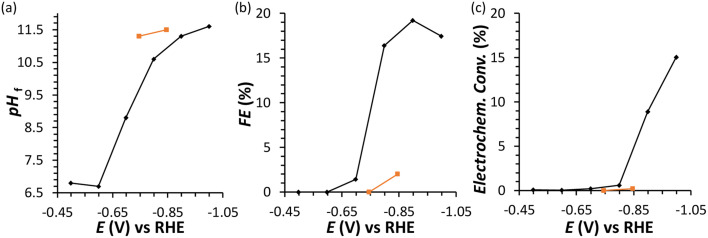
Chronoamperometric FRR tests: final pH (a), Faradaic efficiency (FE) (b), and electrochemical conversion (c) after 1 h electrolysis at different potentials in the range −0.5 to −1.0 V *vs.* RHE. Conditions: a Cu wire was used as WE in a solution with 0.1 M fructose in 0.1 M Na_2_SO_4_ at pH_initial_ = 6.8, room temperature (21 °C), with a Nafion membrane separator. In orange, experiments performed in the presence of 0.1 M glucose instead of fructose.

Linear sweep voltammetry (LSV) was carried out in the potential range between 0.5 and −1.6 V *vs.* RHE in the absence of fructose and in 0.1 M fructose solution, to determine the overpotential needed for the FRR to proceed over the Cu-wire electrode (Fig. 1a). For comparison purposes, the electroreduction of glucose was assessed under the same conditions (Fig. 1a, orange). When comparing the shape of the LSV curves in Na_2_SO_4_ in the absence and presence of fructose (Fig. 1a), no substantial difference can be observed. The same onset potential was identified (*η* = −0.55 V *vs.*RHE), and the overpotential at each current density was only slightly lower in the presence of fructose. The behaviour was very similar when glucose was used instead of fructose (Fig. 1a). The continuous evolution of bubbles was observed in all these tests (see Fig. S20†), indicating the formation of H_2_. These results suggest that the reduction of the monosaccharides is either not occurring or competing with the HER under these conditions. To distinguish between the two hypotheses, a set of experiments was performed in the same electrolyte (0.1 M Na_2_SO_4_, pH = 6.8) and at *E*_appl._ = −0.5 to −1.0 V *vs.* RHE for 1 h (see Fig. 2 and Table S1†), and the concentration of organic species was monitored *via* high-pressure liquid chromatography (HPLC; see Fig. S3 and S4† and Experimental section for HPLC sample preparation and quantification of species). The formation of sorbitol and mannitol as products from the electroreduction of fructose and glucose was observed at |*E*_appl_| > −0.65 V *vs.* RHE, thus demonstrating that the monosaccharide reduction reaction was taking place under such conditions. The fact that the reduction of either fructose or glucose happens in the same potential range and thus competes with the hydrogen evolution reaction is a result of a common first reaction step (Volmer step), which involves the formation of a metal-hydride species derived from the electroreduction of water on the surface of the catalyst (H_2_O + e^−^ + M → M–H + OH^−^).^18^

In an initial chronoamperometric study of the FRR, it was observed that the final pH in the cathodic compartment increased (and decreased in the anodic compartment), and that this phenomenon was dependent on the applied potential (Fig. 2a; see also Fig. S2† for cell scheme and compartments). This happens as a result of the consumption of H^+^ or production of OH^−^ (depending on the pH of the reaction medium) in the cathodic compartment, see eqn (1) and (2). Initially, the pH of the 0.1 M Na_2_SO_4_ electrolyte solution is slightly acidic (pH = 6.8), which means that the FRR and HER should proceed through consumption of H^+^ species. At the same time, the OER in the anodic compartment generates H^+^, as shown in eqn (3). Ideally, the [H^+^] in the two compartments should be kept equal by the transfer of H^+^ from the anodic to the cathodic compartment through the Nafion ion-exchange membrane. However, due to the low concentration of H^+^ in the solution ([H^+^] = 1.6 × 10^−7^ M at pH = 6.8), the Na^+^ in the electrolyte solution is more likely to be the species transferred to maintain the electrical neutrality in the two compartments due to its much higher concentration in the electrolyte solution ([Na^+^] = 0.2 M in 0.1 M Na_2_SO_4_). This would explain the observed trend of gradual increase of the pH in the cathodic compartment and the gradual decrease in the anodic compartment. At the same time, an increase in the Faradaic efficiency towards the conversion of fructose was observed in the experiments performed at |*E*_appl._| ≥ −0.65 V *vs.* RHE (Fig. 2b). This trend of increasing Faradaic efficiency is mainly attributed to the observed change of the pH (Fig. 2a). This behaviour can be rationalised considering that the rate of the mutarotation of fructose is expected to increase at more basic pH, thus leading to a higher concentration of the linear, electroactive form, and thus facilitating its electrochemical reduction over the competing HER. This phenomenon is particularly noticeable when the pH exceeds 10.5. It should be noted that the increase of pH does not only promote the desired mutarotation of fructose but also its isomerisation to glucose and mannose (Scheme 1). The isomerisation is undesirable because the electrochemical reduction of glucose is sluggish compared to that of fructose (*vide supra*) and because mannose would generate mannitol rather than sorbitol upon reduction.

Chronoamperometric experiments performed under similar conditions with glucose as the substrate (Fig. 2, orange) showed a lower electrochemical conversion to sorbitol (maximum 0.2% at −0.85 V), and thus a much lower Faradaic efficiency than the experiments with fructose (*ca.* 2% *vs.* 15–20%). These results confirm the anticipated higher reactivity of fructose under the employed conditions, which is attributed to the faster mutarotation to the linear species compared to glucose (for more information on the chemistry of saccharides, see Section S2 in the ESI†).

Inspired by the enhanced performance observed under alkaline conditions, we continued our study by carrying out the reduction of fructose directly in alkaline media, by screening two electrolyte solutions with basic pH. The first electrolyte solution that was investigated was a 0.5 M sodium carbonate buffer solution (CBS) at pH = 10.6 (ionic strength, *I* = 1.25 M), which was identified as the pH at which the Faradaic efficiency towards the FRR was significantly enhanced compared to lower pH (Fig. 2). The second electrolyte solution that was used consisted of a 0.12 M sodium phosphate buffer (PBS; *I* = 0.5 M) at pH = 11.3. This pH was chosen considering that alkalinity boosts the rate of the mutarotation between the cyclic and the linear, electroactive form of fructose, without being so high as to promote the unwanted chemical isomerisation and degradation of fructose (the rate of the latter reactions increases at pH > 11.3, as we showed in control tests summarised in Section S3 of the ESI, Fig. S13a and b†). In both cases, a buffering solution was chosen in order to keep the pH constant as long as possible (a control test to exclude effect of electrolyte concentration was performed in 0.5 M PBS at pH = 11.3 and showed analogous FE to the test in 0.12 M PBS at the same pH, Table S2†). For comparison with the results with Na_2_SO_4_ at pH = 6.8, an additional experiment was run with PBS at pH = 7.5 (0.18 M; *I* = 0.5 M). Comparing the LSV in the three electrolytes in the presence of fructose (Fig. 1b), we can observe that the absolute value of the onset potential (*vs.* RHE, so pH independent) decreases in the order Na_2_SO_4_ > CBS > PBS (pH_initial_ = 7.5) > PBS (pH_initial_ = 11.3), which indicates that not only the pH but also the type of salt used in the electrolyte solution contributes to determine the potential required to reach a specific current. It is worth noting that, analogously to what was observed in Na_2_SO_4_, in CBS and PBS the LSV curves with and without fructose are rather similar (compare Fig. 1a with Fig. S21†), confirming the competition between the HER and FRR.

**Fig. 3:**
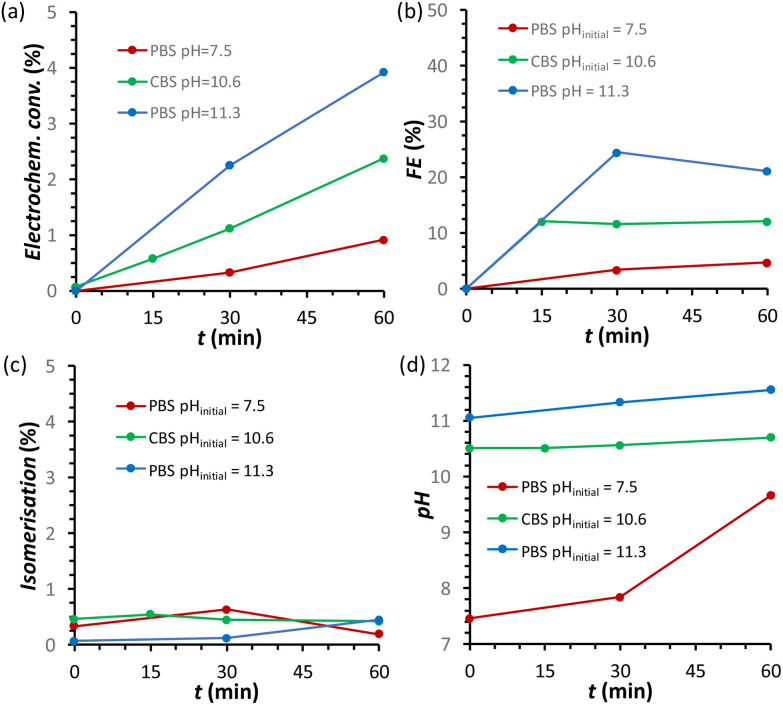
Chronopotentiometric study of FRR on a Cu-wire electrode as a function of the type of electrolyte: (i) 0.18 M PBS at pH = 7.5 (maroon); (ii) 0.5 M CBS pH_initial_ = 10.6 (green); and (iii) 0.12 M PBS pH_initial_ = 11.3 (blue). The following quantities are reported as a function of the electrolysis time: (a) electrochemical conversion; (b) Faradaic efficiency for FRR; (c) isomerisation degree; (d) pH in the cathodic compartment. Conditions: 0.1 M fructose, constant current *i* = −10 mA (*j* = −2.65 mA cm^−2^), room temperature (21 °C), with a Nafion membrane separator.

**Fig. 4:**
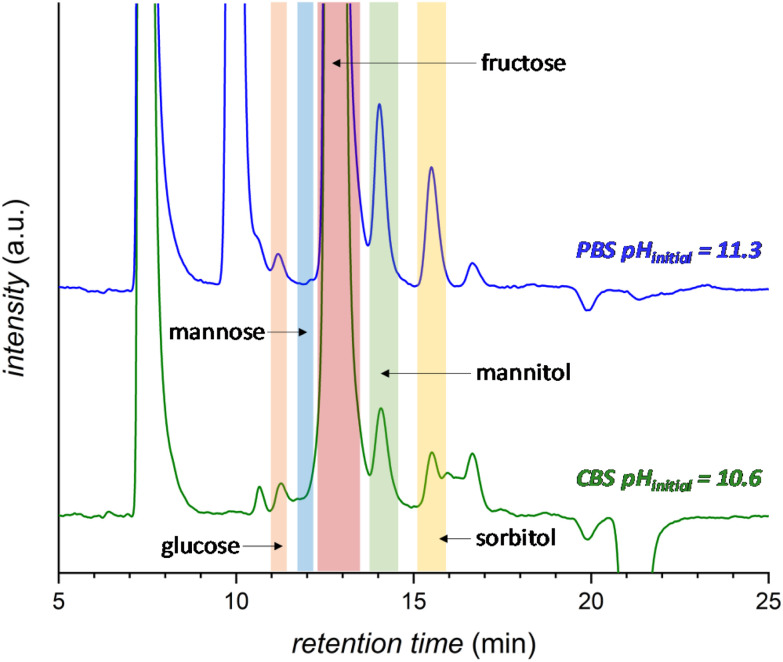
HPLC chromatogram after 1 h electrochemical reduction of fructose at room temperature (21 °C) in 0.5 M CBS (blue) at pH_initial_ = 10.6 or 0.12 M PBS (green) at pH_initial_ = 11.3. The peak at 7.5 min corresponds to the H_2_SO_4_ eluent and the peak at 10 min to the PBS. Possible degradation species (see Fig. S3† for further information): glycolic acid (*t* = 15.9 min), formic acid (*t* = 16.2 min) and acetic acid (*t* = 17.3 min).

The effect of the pH of the electrolyte solution was investigated further by chronopotentiometry (Fig. 3) at a current of *i* = −10 mA, corresponding to a current density of *j* = −2.65 mA cm^−2^. We chose to perform these electrolysis experiments in galvanostatic mode (*i.e.* at a fixed current) rather than in potentiostatic mode because at a fixed current density the sum of all the electrochemical conversions (mainly the FRR and HER) will be the same in different experiments, making it easier to compare Faradaic efficiency results. The chronopotentiometry experiments with an initial pH of 7.5, 10.6 and 11.3 showed that the FE (after 1 h of electrolysis) can be enhanced from 4.7 to 21.1% (based on sorbitol + mannitol yields) by simply increasing the initial pH of the electrolyte from 7.5 to 11.3 (Fig. 3b and Fig. S5 and Table S2†). The improved Faradaic efficiency can be correlated to the increase in electrochemical conversion observed by increasing the initial pH of the electrolyte solution (Fig. 3a). In addition, the buffer properties of both CBS and PBS limit the previously described increase of pH in the cathodic compartment (Fig. 3d), particularly for the buffers with higher starting pH. As a consequence, the undesirable competition of the isomerisation of fructose is minimised and kept below 1% (Fig. 3c). Based on HPLC analysis after 1 h of chronopotentiometry (see Fig. 4 for a representative chromatogram), the expected formation of sorbitol and mannitol (in an average ratio 0.43 : 0.57, see Table S2†) as products of the reduction of fructose was confirmed, with higher yields in the case of the PBS electrolyte with pH_initial_ = 11.3 as a result of the enhanced Faradaic efficiency. Additionally, small peaks originating from the isomerisation (glucose, mannose) and, especially in the presence of CBS, from the degradation (presumably: formic, acetic and glycolic acids, see also Fig. S3†) of fructose were observed.

In order to minimise the competing HER and achieve higher Faradaic efficiency towards the FRR, the cell and reaction parameters were studied and optimised in the electrolyte that gave the best results in the previous tests (*i.e.* 0.12 M PBS at pH = 11.3). Considering these alkaline conditions, Nafion did not seem to be the most suitable option to ensure the transport of ions between the cathodic and anodic compartments and a stable pH. Therefore, we used anion-exchange membranes to try improving the OH^−^ transfer from the cathodic to the anodic compartment and thus to maintain a constant pH. AMVN and AHO (Forblue™ Selemion) membranes were tested at *i* = −10 mA in 0.12 M PBS at pH = 11.3 (see Fig. S6†) but no improvement on the electrochemical conversion or Faradaic efficiency was achieved. Only the increase on the pH became less pronounced with the AMVN membrane, and for this reason this membrane was chosen for the follow-up studies. Next, we studied the role of current density on the FRR efficiency in order to understand whether higher current (and thus applied potential) would have an effect on the FRR:HER competition. By varying the cell current from *i* = −2 to −5, −10 and −20 mA, the electrochemical conversion and, consequently, the final pH in the cathodic compartment increased (up to 11.8 after 1 h at *i* = −20 mA) with the increase of the current (and thus of the number of electrons exchanged in the chosen reaction time), but the FE (%) was not significantly affected, with a maximum of FE = 23% after 1 h (Fig. S7 and Table S3†). Considering this lack of significant influence of the current on the Faradaic efficiency towards the FRR, all the following experiments were performed at a fixed current of *i* = −20 mA (*j* = −5.3 mA cm^−2^), to ensure high conversion degrees and thus facilitate the analysis of the reaction products *via* HPLC. Another factor that may influence the competition between the FRR and HER is the reaction temperature. Increasing the temperature was shown to shift the glucose–fructose equilibrium towards the latter, with the conversion between the two proceeding through the formation of the linear glucose and fructose species.^31^ Therefore, temperature may affect the ratio of the cyclic and linear forms of fructose, of which the latter is the electroactive species. However, temperature is expected to affect (also) the rate of isomerisation and degradation of fructose. Indeed, chemical conversion studies (Section S3†) showed that increasing the temperature from 21 to 45 °C enhanced the degree of isomerisation and degradation of fructose, while carrying out the experiments at 30 °C allowed to keep these chemical reactions at low conversions (Fig. S13†). Hence, we decided to compare the electrochemical conversion of fructose (only) at 21 and 30 °C (Fig. S8† and Table 1). After 3 h of reaction, no positive effect was found on the Faradaic efficiency by carrying out the experiment at 30 °C, while the degree of isomerisation to glucose and mannose was 3 times higher than at room temperature (21 °C). Moreover, a faster decrease of the FE (%) was observed at 30 °C, corresponding to a lower final conversion to sorbitol and mannitol, indicating that 21 °C is a more suitable temperature to avoid side reactions and to maintain a higher Faradaic efficiency.

**Table 1:** Summary of the results for the electrocatalytic reduction of 0.1 M fructose on a Cu-wire electrode in a 0.12 M PBS solution at pH_initial_ = 11.3 at room temperature (maroon) and 30 °C (green) at fixed *i* = −10 mA (*j* = −2.65 mA cm^−2^), with an AMVN membrane. Note that the initial pH of the PBS decreases from 11.3 to 10.3 upon increasing the temperature to 30 °C

*T* (°C)	Time (h)	pH	Isomerisation (%)	Electrochem. conv. (%)	Sorbitol selectivity (%)	FE towards FRR (%)
21	0.5	11.3	0.35	1.8	50	20
	1	11.5	0.40	3.2	50	17
	3	12.0	2.33	5.5	48	10
30	0.5	10.5	1.04	2.2	54	25
	1	10.7	1.89	2.6	32	14
	3	11.1	7.11	3.9	40	7

### Control of pH during the FRR

**Fig. 5:**
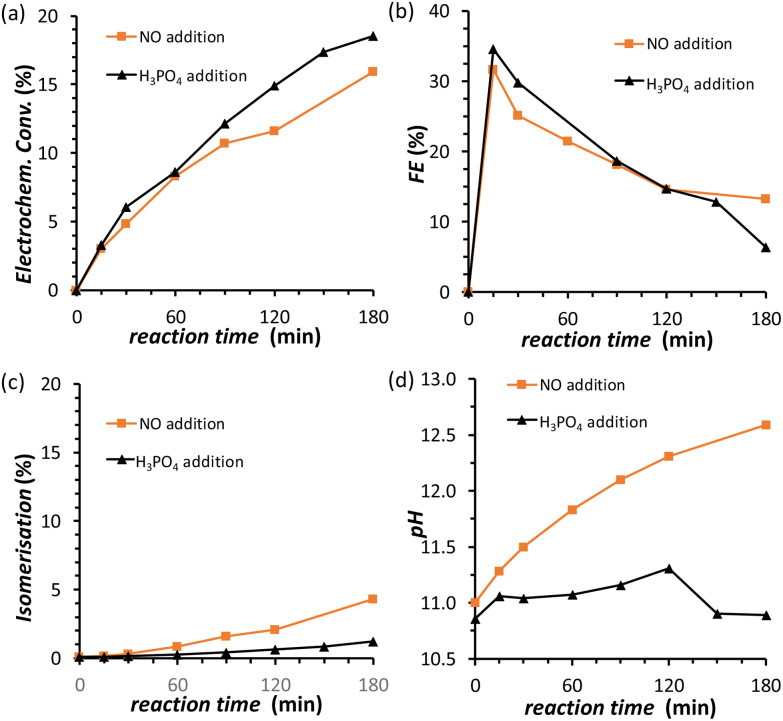
Chronopotentiometric study of FRR on a Cu-wire electrode in a 0.12 M PBS solution at pH_initial_ = 11.3, with the addition (black) of 0.5 M H_3_PO_4_ as titration solution. The following quantities are reported as a function of the electrolysis time: (a) electrochemical conversion; (b) Faradaic efficiency for FRR; (c) isomerisation degree; (d) pH in the cathodic compartment. Conditions: *i* = −20 mA (*j* = −5.3 mA cm^−2^), AMVN membrane separator, room temperature (21 °C).

The results discussed so far demonstrated that to achieve the efficient electrochemical reduction of fructose to sorbitol and mannitol, it is crucial to maintain the pH of the electrolyte solution sufficiently high as to promote the rate of the mutarotation of fructose while avoiding pH > 12 to minimise the undesired chemical side reactions of fructose (isomerisation and degradation). Therefore, in order to minimise the variation of pH as the reaction proceeds, a titration pump was used to maintain the pH in the cathodic compartment at a stable value for the whole experiment (see Fig. S2† for a picture of the set-up). The pump operated with a 0.5 M H_3_PO_4_ aqueous solution (*I* = 0.5 M) in order to maintain the ionic strength and type of species present in the reactor. An addition rate of 10 μL min^−1^ was found to allow preserving a nearly constant pH in the range 10.8–11.2 for experiments at *i* = −20 mA (Fig. 5d). The pH stabilisation *via* the use of a titration pump had a positive influence on the FRR as it allowed suppressing largely the chemical conversions (isomerisation and degradation, Fig. 5c) while preserving and even slightly improving the rate of the electrochemical conversion (Fig. 5a and Table S4†). As a consequence, the ratio between the desired FRR and the unwanted chemical conversion was significantly enhanced. A possible downside of this methodology is the constant dilution of the reaction mixture due to the addition of H_3_PO_4_. However, according to the obtained FE (%) values, the dilution of fructose did not affect the competition between FRR and HER in the explored reaction time.^35^ Although the control of the pH proved very beneficial for limiting the chemical side reactions, it did not have any effect on the FE towards the FRR, which gradually decreased as the reaction proceeded with a similar trend regardless of whether the pH was controlled or not (Fig. 5b). Therefore, we set out identifying the reason and finding a solution for the observed decreasing trend in Faradaic efficiency.

### Electrode de-poisoning

**Fig. 6:**
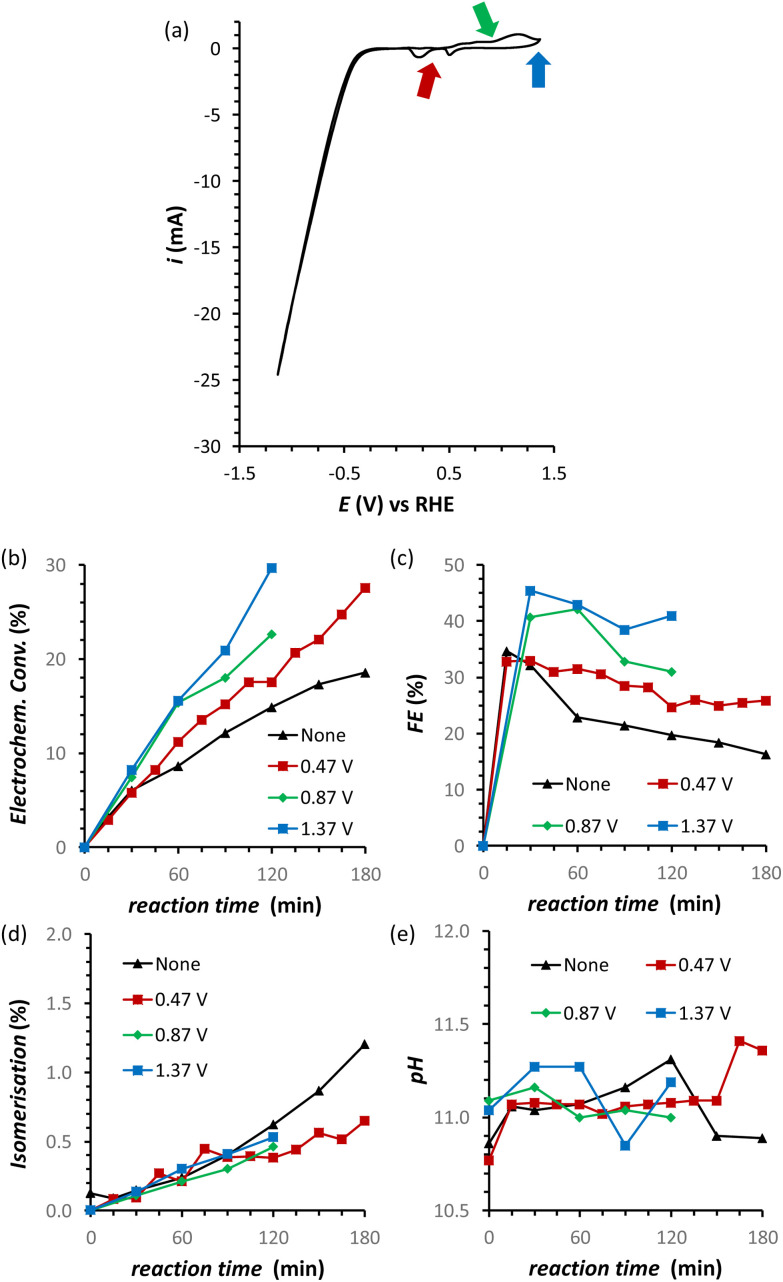
(a) Cyclic Voltammetry (CV) of the Cu-wire electrode at 50 mV s^−1^ scan rate sweeping in a potential window from 1.37 V to −1.3 V *vs.* RHE. (b–d) Effect of oxidative pulses on the FRR on a Cu-wire electrode in a 0.12 M PBS solution at pH_initial_ = 11.3: (b) electrochemical conversion, (c) Faradaic efficiency, (d) isomerisation and (e) the pH. Conditions: *i* = −20 mA (*j* = −5.3 mA cm^−2^), AMVN membrane as separator, constant pH titration with 10 μL min^−1^ of 0.5 M H_3_PO_4_, 3 h at room temperature (21 °C).

A possible cause of the observed drop in Faradaic efficiency over prolonged reaction time (Fig. 5b) is the poisoning of the electrode surface by the substrate and/or reaction intermediates and products. During the electrocatalytic reaction, the adsorption of fructose on the Cu electrode surface is a required step for the following reduction. However, both the substrate and the products could remain adsorbed on the surface of the electrode during the reaction. This poisoning phenomenon has been proposed to occur during the oxidation of saccharides,^36–38^ as well as in the electrocatalytic reduction of fructose in a membrane flow-cell reactor.^21^ Inspired by strategies adopted to overcome this type of poisoning during the oxidation of glucose,^11^ we investigated the use of oxidative pulses at different potentials as a way to partially or totally passivate the surface of the Cu electrode and/or desorb the species from the electrode surface.^38,39^ Based on these studies, it can be proposed (a) that the adsorption of substrate, reaction intermediates, products and by-products is enhanced when the metal electrode is in its metallic state, and (b) that applying an oxidative pulse to oxidise the metal surface would desorb the species from the surface of the electrode, allowing the substrate (being present in higher concentration) to adsorb again when the reductive potential is applied and the electrode is reduced back to its metallic state. Under the reductive conditions in this work (*E* < −1.8 V *vs.* Ag/AgCl *i.e. E* < −0.93 V *vs.* RHE), Cu should be present only in the metallic state according to the Pourbaix diagram (Fig. S14†) and voltammetric studies (see Section S4 in the ESI†), which supports the hypothesis that poisoning species accumulate on the electrode surface and that the oxidative pulse promotes their desorption. The effect of the oxidative pulses as a de-poisoning strategy was investigated during the chronopotentiometry experiments at *i* = −20 mA, by applying an oxidative pulse for 5 s every 5 min (Fig. 6 and Table S5†). The potential at which the oxidative pulses were carried out was also screened. Three potentials were chosen based on an analysis of the Cu-wire electrode by cyclic voltammetry (Fig. 6a and Fig. S16, S17†): (1) 0.47 V (*vs.* RHE; −0.2 V *vs.* SHE, red arrow in Fig. 6a) was selected as potential at which no Cu oxidation takes place, in order to determine whether the switch to a non-Faradaic current would be enough to re-activate the Cu electrocatalyst; (2) 0.87 V (0.2 V *vs.* SHE, green arrow in Fig. 6a) was chosen being a potential that is sufficiently oxidative to lead to the 1e^−^ oxidation to Cu_2_O but not so high that it would cause further Cu oxidation; and (3) 1.37 V (0.7 V *vs.* SHE, blue arrow in Fig. 6a) was chosen as an extremely high potential to make sure Cu was fully oxidised to CuO, which is expected to grant the most effective removal of adsorbed poisoning species.

**Fig. 7:**
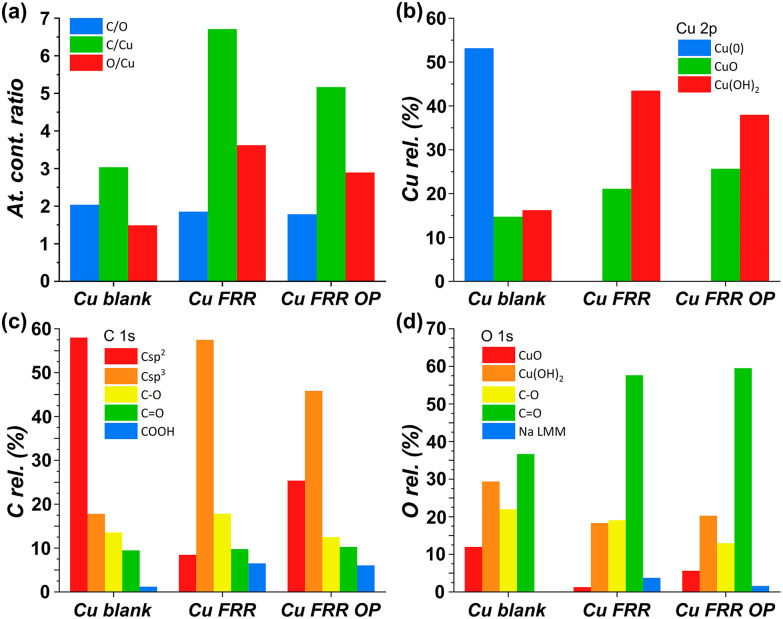
XPS analysis of different electrodes: Cu blank (pristine Cu electrode), Cu FRR (electrode after 10 h electrolysis at *i* = −20 mA), and Cu FRR OP (electrode after 10 h electrolysis at *i* = −20 mA including 5 s oxidative pulses at 1.37 V *vs.* RHE every 5 min). Conditions: 0.1 M fructose, AMVN membrane as separator, constant pH titration with 10 μL min^−1^ of 0.5 M H_3_PO_4_, room temperature (21 °C). (a) Ratios between atomic contents (C, O and Cu); (b) relative content (%) of Cu species from Cu 2p core levels; (c) relative content (%) of C species from C 1s core level; and (d) relative content (%) of O species from O 1s core level (Na LMM is the Auger line of sodium, which overlaps with the O 1s signal).

Our strategy proved successful and a substantial difference on the performance of the Cu electrocatalyst in the FRR was observed when applying the oxidative pulses (Fig. 6: red, green and blue lines) compared to the experiment without pulses (black). A positive effect on both electrochemical conversion and Faradaic efficiency was observed already with pulses at 0.47 V *vs.* RHE (red), and this effect was enhanced at higher applied oxidative potentials (Fig. 6a and b), reaching a FE > 40% and 30% electrochemical conversion within 2 h. Besides promoting the reactivation of the electrode, the oxidative pulses can also cause leaching of Cu species from the electrode. Therefore, the influence of the 1.37 V *vs.* RHE oxidative pulse on the stability of the Cu electrode was studied by ICP-OES analysis of the electrolyte solution (Table S6†). These data suggest that the oxidative pulses induced minor Cu leaching (Fig. S10 and Table S6 entries 5–8†), although the concentration is in the range of the equipment detection limit (0.4 ppm detected *vs.* 0.1 ppm before the oxidative potential). After applying the reductive catalytic potential again for 5 min (Table S6, entry 7†), the concentration of Cu in solution was unaffected, which suggests that the leached Cu was not reduced back to the metallic state. However, since the amount of dissolved Cu is so close to the ICP-OES detection limit, caution is advised in the interpretation of these data. The investigation of the effect of the oxidative pulses on the Cu electrode was deepened by characterisation of the surface composition of the electrode using X-ray photoelectron spectroscopy (XPS, Fig. 7 and Fig. S10–S12 and Table S7†). These measurements were performed on Cu plates (S.A. = 4.725 cm^2^) instead of wires. A blank sample of a Cu-plate (Cu blank) was compared to a sample after 10 h electrolysis at *i* = −20 mA (Cu FRR) and another sample at the same experimental conditions but including 5 s oxidative pulses at 1.37 V *vs.* RHE every 5 min of electrolysis (Cu FRR OP). The analysis revealed an increase of the content of C and O relative to the content of Cu, after the Cu electrode was utilised in the FRR (Cu FRR) compared to the pristine electrode (Cu blank), see Fig. 7a. This result suggests the adsorption of fructose and/or intermediates and products on the electrode surface. The C and O contents on the pristine commercial Cu electrode are attributed to the native oxide layer (*i.e.* Cu(OH)_2_ and CuO) and to a polymeric coating that is present at the surface of the Cu plate (see Fig. S10 and S11 and the related text in the ESI†). When the oxidative pulses were applied during the FRR tests, the C/Cu and O/Cu ratios were lower compared to the tests without pulses, while the C/O ratio was similar in the two cases (Cu FRR OP *vs.* Cu FRR in Fig. 7a). These results are in agreement with the anticipated presence of adsorbed organic species at the electrode surface during the FRR experiments, and with their partial removal upon application of the oxidative pulses (*vide supra*). The oxidative pulses also had an effect on the Cu species: the pristine Cu electrode (Cu blank) was characterised by a metallic core (Cu^0^) and a CuO/Cu(OH)_2_ surface layer (Fig. 7b). The oxidised Cu species became predominant after the electrode was employed in the FRR tests (Cu FRR and Cu FRR OP, see Fig. 7b and S11a,b, S12a†), as a consequence of interaction with the aqueous electrolyte. Notably, with the application of the oxidative pulses, the relative content of CuO slightly increased while that of Cu(OH)_2_ decreased. The analysis of the O 1s and C 1s core levels (Fig. 7c and d and Fig. S12b,c†) when the electrode was subjected to the 1.37 V *vs.* RHE oxidative pulse (Cu FRR *vs.* Cu FRR OP) revealed a decrease of the C sp^3^ (C–C) and an increase of the C sp^2^ (C=C) components, a constant relative content of C=O and a decrease of C–O functionalities. These observations confirm the higher degree of poisoning by adsorbed species in Cu FRR (containing C–C and C–O, in combination with Cu(OH)_2_ surface species) compared to Cu FRR OP, and suggest that the oxidative pulses mainly remove the reduced species (C–O, C–C), thus helping restore the electrocatalytic performance of the Cu electrode.

X-ray diffraction (XRD) analysis of the Cu blank, Cu FRR and Cu FRR OP electrodes showed in all the cases only one crystalline phase corresponding to metallic Cu (Fig. S9†). This indicates that the oxidised Cu species observed in the XPS analysis correspond to an amorphous thin layer of Cu oxide/hydroxides at the surface of the copper plate.

### Long FRR tests

**Fig. 8:**
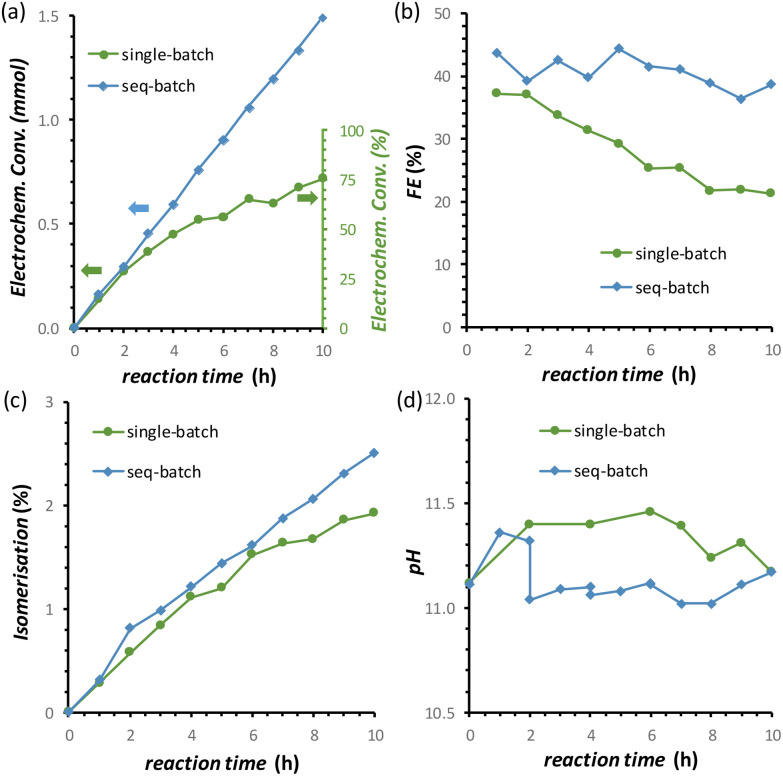
Effect of performing a long (10 h) chronopotentiometric FRR test on a Cu-wire electrode in a single-batch approach with a 0.1 M fructose electrolyte solution (green) or with a sequential-batch approach in which the 0.1 M fructose electrolyte solution was restocked every 2 h (blue) on: (a) electrochemical conversion; (b) Faradaic efficiency; (c) isomerisation degree; (d) the pH. Conditions: *i* = −20 mA (*j* = −5.3 mA cm^−2^) in a 0.12 M PBS solution at pH_initial_ = 11.3, with the addition of 0.5 M H_3_PO_4_ at 10 μL min^−1^ and by applying a 1.37 V *vs.* RHE oxidative pulse for 5 s every 5 min, AMVN membrane as separator, room temperature (21 °C).

By combining the major improvements in the FFR performance that were achieved both by using the titration pump and by introducing the oxidative pulses, we were able to reach a remarkable 77% electrochemical conversion of fructose to sorbitol and mannitol (obtained in a ratio of 0.43 : 0.57) after 10 h of chronopotentiometric test at room temperature (Fig. 8a, data in green). However, in this experiment, a gradual, slow decrease in the rate of the electrochemical conversion and in the FE (%) was observed (Fig. 8a and b, data in green). We hypothesised that this was not caused by a deactivation of the Cu electrocatalyst but was simply due to the decrease of the concentration of fructose in the solution, which would affect both the reaction rate and FE unless the reaction was zero order in fructose. In order to test our hypothesis, we carried out a 10 h FRR test in which the reaction solution was changed with a fresh one every two hours (sequential-batch test), and compared it with the same experiment without changing the solution (single-batch test). In both tests, the pH remained in the expected range between 11.0 and 11.5 (Fig. 8d). Notably, in the sequential-batch approach the reaction rate remained constant, leading to a steady increase in the cumulative conversion of fructose and to a constant Faradaic efficiency (Fig. 8a and b, data in blue). Meanwhile, in the sequential-batch approach also the rate of the isomerisation remained constant but the total degree of isomerisation stayed low (2.6%, Fig. 8c). These results clearly demonstrate that no deactivation of the Cu electrocatalyst occurred during 10 h of FRR under the optimised conditions developed throughout this study and strongly support our hypothesis that the decrease in reaction rate and FE observed in the single-batch test was a mere consequence of the decrease of fructose concentration in solution. These results are promising for the prospect of achieving the continuous electrochemical reduction of fructose to sorbitol (and mannitol) in a flow-cell reactor under optimised conditions. This would intrinsically allow to tackle (1) the pH problem and (2) the decrease in the fructose concentration. Additionally, (3) the residence time of the substrate and products in the reactor would decrease, allowing to quench the chemical reactions at an earlier stage and thus (4) increase the ratio between electrochemical and isomerisation products.

## Conclusions

In this work, an electrocatalytic system for the electrochemical reduction of fructose to sorbitol and mannitol has been designed and developed using a Cu wire as the electrocatalytic material. A thorough study of the reaction parameters allowed limiting the competition with the HER, increasing the Faradaic efficiency up to 40% towards the formation of sorbitol and mannitol and achieving an unsurpassed 77% electrochemical conversion of fructose while minimising the unwanted isomerisation side reaction (2%) after 10 h at room temperature. These major improvements represent an important green advance towards the conversion of a biobased, abundant compound such as fructose into useful products (sorbitol and mannitol) by using renewable electricity to drive the reaction or potentially co-generating electricity, depending on the anodic reaction coupled to the reduction of fructose. A number of tailored solutions were identified in this work to enable these results. The control of the reaction temperature and pH allowed limiting the chemical side reactions towards glucose, mannose and degradation compounds. A pulsed potential method was developed to achieve the reactivation of the catalyst contributing to preserving the optimum Faradaic efficiency. Finally, using a sequential-batch approach we demonstrated the stability of the electrocatalyst in a long chronopotentiometric run. Compared to the few electrochemical systems previously reported for this reaction, our work has the advantage of using an abundant and cheap metal (namely Cu) as the electrocatalyst, compared to the Pt/Rh electrocatalyst reported in the literature,^20,26^ while also providing a much deeper understanding and control of the crucial parameters that govern the electrocatalytic reduction of fructose. Although Cu proved to be a promising electrode for the FRR, it is not necessarily the optimum choice and future work will aim at investigating and enhancing the features of the electrocatalyst used for this reaction. Additionally, this work also offers an insight into the differences and limitations of using glucose or fructose as the substrate for the electrocatalytic production of sorbitol. Therefore, we believe that this work is relevant not only towards the practical realisation of the fructose reduction reaction, but more in general for the electrochemical upgrading of saccharides and related biobased compounds.

## Experimental section

### Materials

All chemicals were used as received from the supplier. The following chemicals were purchased from Sigma-Aldrich: dimethyl sulphoxide 99.9% (DMSO), sulphuric acid 95.0–98.0% (H_2_SO_4_), anhydrous sodium sulphate ≥99.0% (Na_2_SO_4_), sodium phosphate dibasic heptahydrate ≥99.99% (Na_2_HPO_4_·7H_2_O), sodium bicarbonate ≥99.7% (NaHCO_3_), sodium carbonate ≥99.9% (Na_2_CO_3_), sodium hydroxide ≥98% (NaOH), d-(+)-glucose ≥99.5%, d-sorbitol ≥98%, and d-(+)-mannose ≥99%. Sodium phosphate monobasic dihydrate (NaH_2_PO_4_·2H_2_O) and d-(−)-fructose 99% were purchased from Acros Organics. *ortho*-Phosphoric acid 85% (H_3_PO_4_) was purchased from Supelco. Pure N_2_ gas ≥99.999% (Azote 5.0 Instrument) was purchased from Linde. The electrolyte solutions used in this work were prepared using Milli-Q water according to the following procedures:

• 3443 ppm DMSO solution was prepared by mixing 172.0 mg of DMSO with 49.7842 g of Milli-Q water.

• 0.5 M sulphuric acid solution (H_2_SO_4_) was prepared by diluting 5.132 g of pure H_2_SO_4_ with Milli-Q water to 100 mL.

• 0.53 M phosphoric acid solution (H_3_PO_4_, *I* = 2.5 M, pH = 1.14) was prepared by diluting 5.7436 g of H_3_PO_4_ with 94.55 g of Milli-Q water.

• 0.10 M sodium sulphate solution (Na_2_SO_4_, *I* = 0.25 M, pH = 6.8) was prepared by mixing 7.102 g of Na_2_SO_4_ anhydrous with 100 mL of Milli-Q water.

• 0.18 M sodium phosphate buffer solution (PBS, *I* = 0.5 M, pH = 7.5) was prepared by mixing 390 mg of NaH_2_PO_4_·2H_2_O and 4.209 g of Na_2_HPO_4_·7H_2_O in 100 mL of Milli-Q water.

• 0.50 M sodium carbonate buffer solution (CBS, *I* = 1.25 M, pH = 10.6) was prepared by mixing 0.761 g of NaHCO_3_ and 4.340 g of Na_2_CO_3_ in 100 mL of Milli-Q water.

• 0.12 M sodium phosphate buffer solution (PBS, *I* = 0.5 M, pH = 11.3) was prepared by mixing 3.190 g of Na_2_HPO_4_·7H_2_O with 90 mL of Milli-Q water. The pH was adjusted to 11.3 by adding gradually *ca.* 10 mL of 1 M NaOH.

• 0.50 M sodium phosphate buffer solution (PBS, *I* = 2.5 M, pH = 11.3) was prepared by mixing 13.404 g of Na_2_HPO_4_·7H_2_O with 90 mL of Milli-Q water. The pH was adjusted to 11.3 by adding gradually *ca.* 10 mL of 1 M NaOH.

• 1 M NaOH solution was prepared by mixing 2.0 g of NaOH with 50 mL of Milli-Q water.

### Electrochemical measurements

All the electrochemical experiments were performed in a two-compartment in-house designed glass H-cell equipped with a Gamry 1000 Interface potentiostat. The compartments were separated either with a Nafion 115 membrane (Fuel Cell Store) or Forblue™ AMVN (Selemion) membrane. In one specific experiment, a Forblue™ AHO (Selemion) membrane was also used. The membranes were cut in circular shape with a 22 mm diameter and clamped in the 15 mm cell cross-section between the two compartments. 10 mL of electrolyte was placed in each compartment. For the FRR experiments, the cathodic electrolyte contained 0.1 M fructose solution. The solution was degassed before any electrochemical test and the cathodic compartment was kept in an inert environment with a N_2_ gas flow: (1) to avoid any interference of O_2_ gas on the oxidation/passivation of Cu and on the Faradaic efficiency (due to O_2_ reduction), and (2) to avoid any overpressure due to the formation of H_2_ gas in a closed compartment. The formation of (H_2_) bubbles was clearly observed in all the experiments reported in this work and became more intense at higher current densities. The resistance of the system was tested before starting any experiment, being always in the 10–20 Ω range. A 15 cm Cu wire (0.5 mm diameter, 99.999%; Alfa Aesar) was used as the working electrode (WE), of which only 12 cm (±0.2 cm) was manually coiled around a plastic cylinder of *ca.* 4 mm diameter and then dipped into the reaction solution (S.A. = 3.78 cm^2^), see Fig. S2.† The remaining 3 cm of the Cu wire was used to clamp the electrode to the potentiostat cables. Before each electrochemical experiment, the Cu wire was sonicated in EtOH and Milli-Q water (2×) for 10 min each, in order to remove any grease that might have been present at the surface. For XRD and XPS experiments, a Cu plate (0.5 mm thickness; 99.98%; Sigma Aldrich) was cut in 1.5 cm × 1.5 cm plates (S.A. = 4.725 cm^2^), which were then sonicated in EtOH and Milli-Q water for 10 min each, and employed as WE. A leak-free Ag/AgCl (3.5 M KCl) electrode was used as the reference electrode (RE, Alvatek: LF-1-100) and a graphite rod (6.15 mm diameter; 99.9995%; Alfa Aesar) was used as the counter electrode (CE). In order to exclude the influence of the degradation of graphite (CE) on the reactions in the cathodic compartment, a control test was performed with a Pt-coiled wire electrode, which gave analogous results (Fig. S22†). All potentials were reported *vs.* RHE by adding 0.205 mV (SHE value) and the value corresponding to the Nernst equation: 0.059 × pH, utilising the pH value of the electrolyte before the addition of fructose. All potentials are considered *versus* the reaction of interest (fructose reduction), and thus the more negative the potential, the higher its (absolute) value was considered, unless differently stated. A Mettler-Toledo B.V. Seven2Go pH-meter S2-Std-kit (pH range: −2 to 20; pH resolution: 0.01) was used for monitoring the pH, after calibration using the supplied buffers at pH 4.00, 7.02 and 10.13 (at *T* = 20 °C). The pH-meter was also employed for monitoring the reaction temperature.

Linear sweep voltammetry (LSV) experiments were performed from *E*_i_ ≈ 0.5 V *vs.* RHE (non-Faradaic region in which Cu is not oxidised) up to *E*_f_ = −1.6 V *vs.* RHE (potential at which a *i* = −20 mA was surpassed; pH-dependent potential). A 10 mV s^−1^ scan rate was used.

Cyclic voltammetry (CV) experiments were run at different potentials depending on the goal of the study, in the range between *E* = 1.37 V *vs.* RHE and *E* = −1.15 V *vs.* RHE. A 50 mV s^−1^ scan rate was used. Four consecutive CV cycles were run in order to study if any difference was derived from the sequential cycling of the system, and to understand the influence of shifting the potential to further oxidative/reductive values to the Cu oxidation/reduction peaks. To study the redox properties of the Cu electrode, the following series of CVs were run:

 • *E*_i_ = 0.47 V; *E*_red_ = 0.07 V; *E*_f_ = 0.47 V

 • *E*_i_ = 0.47 V; *E*_red_ = 0.07 V; *E*_f_ = 0.67 V

 • *E*_i_ = 0.67 V; *E*_red_ = 0.07 V; *E*_f_ = 0.77 V

 • *E*_i_ = 0.77 V; *E*_red_ = 0.07 V; *E*_f_ = 0.97 V

 • *E*_i_ = 0.97 V; *E*_red_ = 0.07 V; *E*_f_ = 1.17 V

 • *E*_i_ = 1.17 V; *E*_red_ = 0.07 V; *E*_f_ = 1.37 V

where *E*_i_ is the initial potential, *E*_red_ is the most reductive potential used in the measurement, and *E*_f_ is the final potential, all reported *vs.* RHE. Before each of these experiments, a LSV was carried out from *E*_i_ = 0.47 V *vs.* RHE to *E*_red_ = 0.07 V *vs.* RHE, in order to make sure that the reductive peaks in the cathodic scan appear only as a result of the oxidation on the preceding anodic scan.

Chronoamperometry (CA) experiments were performed in 0.1 M Na_2_SO_4_ pH = 6.8 solution and at *E*_appl._ = −0.5 to −1.0 V *vs.* RHE (*E*_appl_: potential applied for electrolysis) for 1 h.

Chronopotentiometry (CP) experiments were performed at fixed current intensities of *i* = −2 mA, −5 mA, −10 mA and −20 mA, in order to ensure that a homogeneous charge passed through the system between different tests. The required potential to achieve these currents was monitored for each of the experiments. To monitor the progress of the electrochemical reaction, the current was stopped for few seconds to collect HPLC samples and resumed afterwards. For experiments in which an oxidative pulse was applied, a sequence was set consisting of 5 min of CPs at −20 mA and 5 s CAs at the chosen potential. HPLC samples were collected at the end of the corresponding reductive step and just before the following oxidative pulse.

### Physicochemical characterisation

Inductively coupled plasma optical emission spectrometry (ICP-OES) was performed on an Optima 7000 ICP-OES from PerkinElmer. The samples were prepared by diluting around 2 g of each aqueous solution with 1.2% HNO_3_ up to 25 mL. The calibration was carried out with a commercial calibration mix 9487, which contains copper among other elements. A calibration curve was obtained using 0.5; 1; 5 and 10 ppm. The concentration of copper was calculated by means of the calibration curve. Duplicate measurements were carried out and the average and standard deviations were calculated.

X-ray diffraction (XRD) measurements of the Cu plates (0.5 mm thickness, 1.5 cm × 1.5 cm) were performed on a D8 Advance Bruker diffractometer with a Cu Kα1 radiation (*λ* = 1.5418 Å) under 40 kV and 40 mA in the range of 10–80°.

X-ray photoemission spectroscopy (XPS) analysis was performed using a Surface Science SSX-100 ESCA instrument with a monochromatic Al Kα X-ray source (1486.6 eV). The XPS analysis was performed on a Cu plate (1.5 cm × 1.5 cm) instead of on a Cu wire, to ensure a flat and homogeneous surface and thus to obtain reproducible spots with high detector counts. The pressure was maintained at 10^−9^ mbar with the electron take-off angle of 37°. The XPS data were acquired on a 1000 μm diameter spot with energy resolution set to 1.3 eV (for both the survey spectra and core level regions). Binding energies are reported ±0.1 eV. All XPS spectra were analysed using the curve-fitting program CasaXPS. The deconvolution of the spectra included a Shirley baseline subtraction and fitting with a minimum number of peaks consistent with the sample composition (taking into account the experimental resolution). The profile of the peaks was taken as a convolution of Gaussian and Lorentzian functions [GL(90) for Cu 2p region and GL(30) for the O 1s, C 1s and Si 2p regions]. The uncertainty in the peak intensity determination was within 2% for all core levels reported. All measurements were carried on two distinct spots on each sample in order to check the degree of homogeneity. Relative elemental content [Cu, C or O rel. (%)] was calculated as relative percentage content of the specific element with respect to the total amount of the element in the sample (*e.g.* C sp^3^ rel. (%) = C sp^3^ at%/tot. C at% × 100%). Prior to the electrochemical tests, the Cu plate (1.5 cm × 1.5 cm) was cleaned *via* sonication in ethanol (10 min) and then mQ water (10 min). To assess the presence of a polymeric coating, the Cu plate was polished with SiC paper (4000) and 3 μm diamond paste, and then cleaned as previously described (polished Cu blank). The blank sample of a Cu plate (Cu blank) was compared to a sample after 10 h of electrolysis at *i* = −20 mA (Cu FRR), and another sample at the same experimental conditions and including 5 s oxidative pulses at 1.37 V *vs.* RHE every 5 min of electrolysis (Cu FRR OP). Core level peak assignment and respective binding energies (BE, in eV) are presented in Table S7.†

### HPLC data analysis and product quantification

High-pressure liquid chromatography (HPLC) analysis was performed on an Agilent Technologies 1200 series chromatograph equipped with Bio-Rad Aminex HPX-87H 300 × 7.8 mm column at *T* = 60 °C with 0.5 mM aqueous H_2_SO_4_ eluent (flow rate: 0.55 mL min^−1^) and a refractive index detector. HPLC samples were prepared by diluting 100 μL of the reaction solution with 300 μL of a 3443 ppm DMSO solution, and 50 μL of a 0.5 M H_2_SO_4_ aqueous solution (unless differently stated). H_2_SO_4_ was used in order to quench any chemical reaction (isomerisation or degradation) that would otherwise proceed in the waiting time between the sample preparation and its analysis in the HPLC equipment (this was done in all tests apart from the preliminary chronoamperometry in Fig. 2). The exact amounts were weighed with a precision scale. Calibration of the HPLC chromatograph was performed by analysing samples containing known concentrations of the sugars (glucose, mannose, fructose) and derived products (sorbitol and mannitol) in the presence of DMSO as the internal standard. The calibration stock samples were split in two to avoid signal overlapping: (a) glucose + fructose + sorbitol, and (b) mannose + mannitol (see calibration curves in Fig. S4 in the ESI†).

The mass concentration (*P*_a_, in ppm) of compound a, was obtained from the peak area of the compound (*A*_a_) and of DMSO (*A*_DMSO_) using the slope of the calibration curve (*b*) and considering the specific sample dilution and the known mass concentration of DMSO (*P*_DMSO_, in ppm).
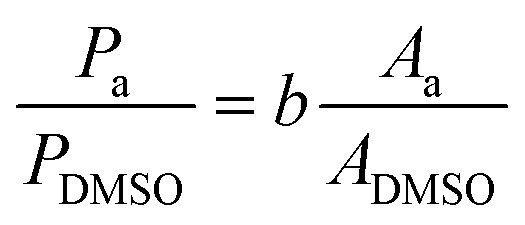


To calculate the number of moles of each species (*n*_a,*x*_) in the reaction solution for each sample (*x*), the initial reaction mass was used, which is the sum of the electrolyte mass (*m*_PBS_) and the substrate mass (*m*_subs._). For each sample (*x*), the sampling mass for all the previous HPLC samples (*x* − 1, *x* − 2…) was subtracted (*S*_*y*_) from the initial mixture mass, resulting in the total mass of the reaction solution at the time that each sample was taken. This value was multiplied by the mass concentration of each compound (*P*_a_) and divided by the molecular mass of the compound (*M*_a_), to give the number of moles of each species (*n*_a,*x*_).
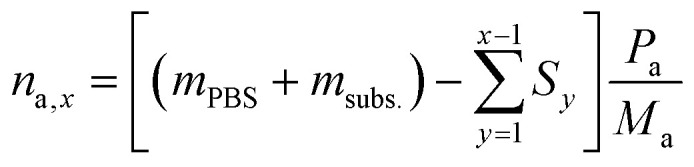


The molar concentration (*M*_a_) at each time was calculated by dividing the number of moles by the remaining water volume. The reaction volume at each time was calculated by using the water mass percentage at time *t* = 0 (%_w_), and multiplying it by the mass of the reaction mixture at each time (PBS + fructose – previous HPLC samplings), and considering the density of water to be *δ* = 1 g mL^−1^.
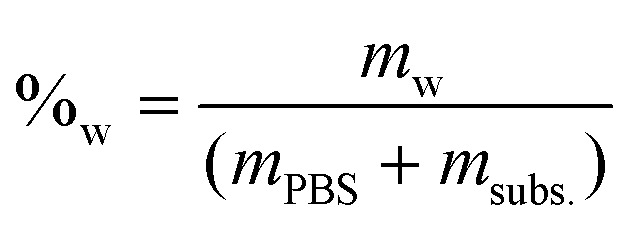

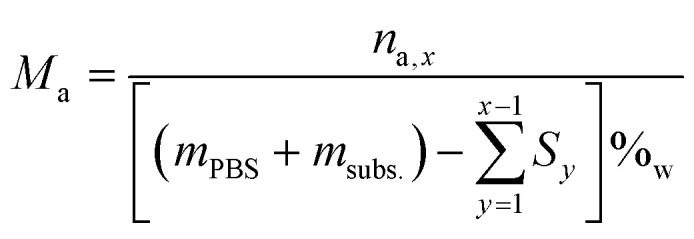


The yield of each compound (Y_a_) was calculated by dividing its molar concentration (*M*_a_) by the initial substrate concentration (*M*_subs.,*t*=0_), and given in percentage.
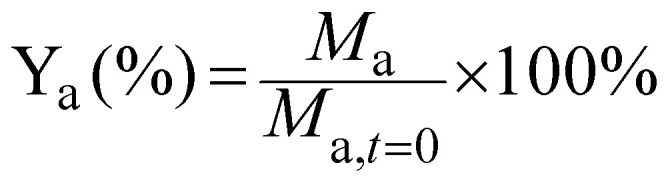


Isomerisation is the chemical conversion of fructose to its stereoisomers glucose and mannose due to the basicity of the reaction medium, and was calculated by adding up the yields of these two compounds. The electrochemical conversion of fructose was calculated as the sum of sorbitol and mannitol yields.

The product selectivity refers to the selectivity between the products of the direct electrochemical reduction of fructose, and it was calculated by dividing the moles of compound a (*n*_a_, sorbitol or mannitol) by the sum of the moles of the two products (sorbitol and mannitol), and given in percentage.

The Faradaic efficiency (FE) was calculated by dividing the number of moles of sorbitol and mannitol by half of the number of moles of electrons that had gone through the electric circuit, and given as a percentage. Half of the moles of electrons were used according to the reduction half-reaction (eqn (1)), in which two electrons are exchanged per every fructose molecule that is converted into one sorbitol/mannitol molecule.
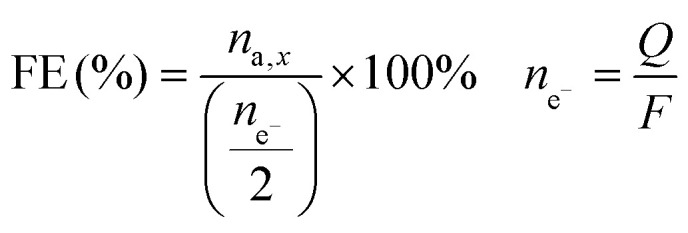


The number of moles of electrons was calculated using Faraday's law, *i.e.* dividing the charge passed through the system (*Q*, expressed in C) by the Faraday constant (*F* = 96 485 C mol^−1^). The FE is thus an indication of the competition between the FRR and the HER.

For the reactions in which H_3_PO_4_ was added to control the solution pH, the theoretical concentration of the initial substrate (*N*_subs.,*x*_) was calculated at every sampling step, to take into account the continuous dilution of substrate and products. To do so, the number of moles that were removed in each sampling step was calculated and subtracted from the initial substrate concentration as follows:
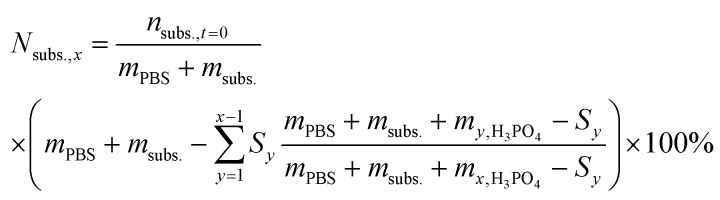
where *m*_*y*,H_3_PO_4__ is the mass of 0.5 M H_3_PO_4_ added between the previous HPLC sample and the current sample, and *m*_*x*,H_3_PO_4__ is the mass of 0.5 M H_3_PO_4_ added in the previous step in the sequence.

From this value, the ratio of initial fructose (*R*_subs.,*x*_) that was present at every sampling step was also calculated.
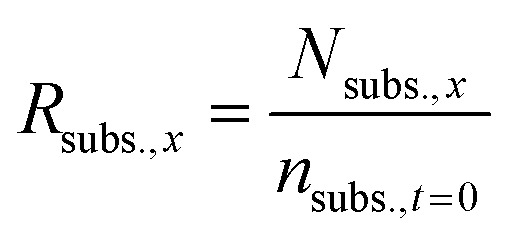


In these cases, the conversion was calculated by dividing the number of moles of each compound found *via* HPLC analysis by the theoretical concentration of initial fructose, thus avoiding underestimation of the reaction conversion. The Faradaic efficiency was calculated by following the method previously described, but in this case dividing also by the ratio of initial fructose (*R*_subs.,*x*_) that was present at every sampling step, thus avoiding underestimation of the reaction conversion.

## Author contributions

Dr. Jordi Creus: conceptualisation, major experimental production, data curation, analysis, methodology, visualisation, writing the original draft, and review and editing. Dr. Matteo Miola: experimental production, data curation, analysis, visualisation, and reviewing and editing of the manuscript. Prof. Dr. Paolo P. Pescarmona: conceptualisation, funding and resources, analysis, methodology, supervision, and reviewing and editing of the manuscript.

## Conflicts of interest

There are no conflicts to declare.

## Supplementary Material

GC-025-D2GC04451J-s001
