# Supplementary material for: Unravelling and overcoming the challenges in the electrocatalytic reduction of fructose to sorbitol
Source: Green Chem. 2023 Jan 12;25(4):1658–71. doi: 10.1039/d2gc04451j (PMC9940304; doi:10.1039/d2gc04451j)
Supplement: GC-025-D2GC04451J-s001 [file GC-025-D2GC04451J-s001.pdf]

Electronic Supplementary Material (ESI) for Green Chemistry.
This journal is © The Royal Society of Chemistry 2023

# Unravelling and overcoming the challenges in the electrocatalytic reduction of fructose to sorbitol

Jordi Creus, Matteo Miola, Paolo P. Pescarmona*

Chemical Engineering Group, Engineering and Technology Institute Groningen (ENTEG), University of Groningen, Nijenborgh 4, 9747 AG Groningen, the Netherlands. E-mail: p.p.pescarmona@rug.nl

## Supporting Information

### Table of contents:

S1. Additional data ....... 2

S1.1 XPS figures ....... 11

S2. The chemistry of saccharides ....... 14

S3. Study of the chemical conversion of fructose ....... 17

S4. Redox properties of the Cu catalyst ....... 19

## S1. Additional data

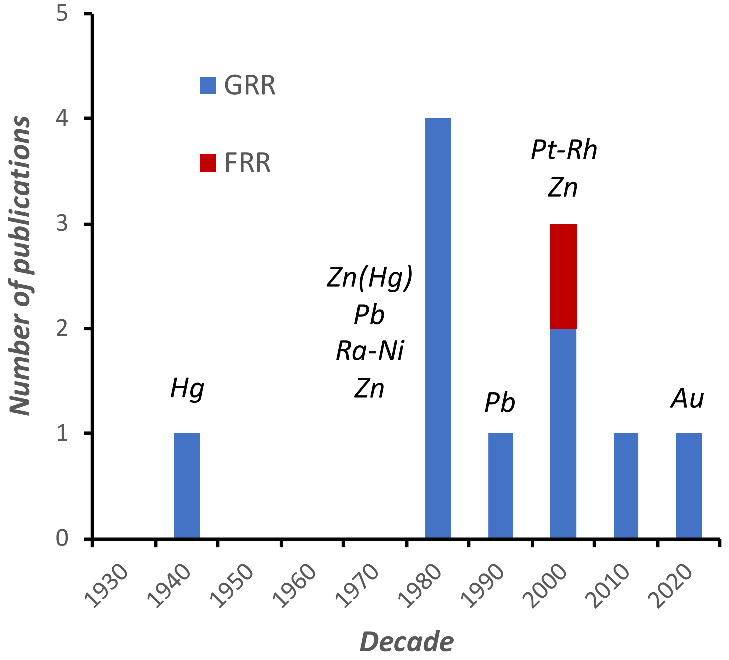


*Figure S1. Number of publications in the field of monosaccharides electrocatalytic reduction, based on the conversion of glucose and other monosaccharides (blue, 10 publications) and fructose (maroon, 1 publication). In the publication in the 2010 decade, a large set of metals was tested.[1]*

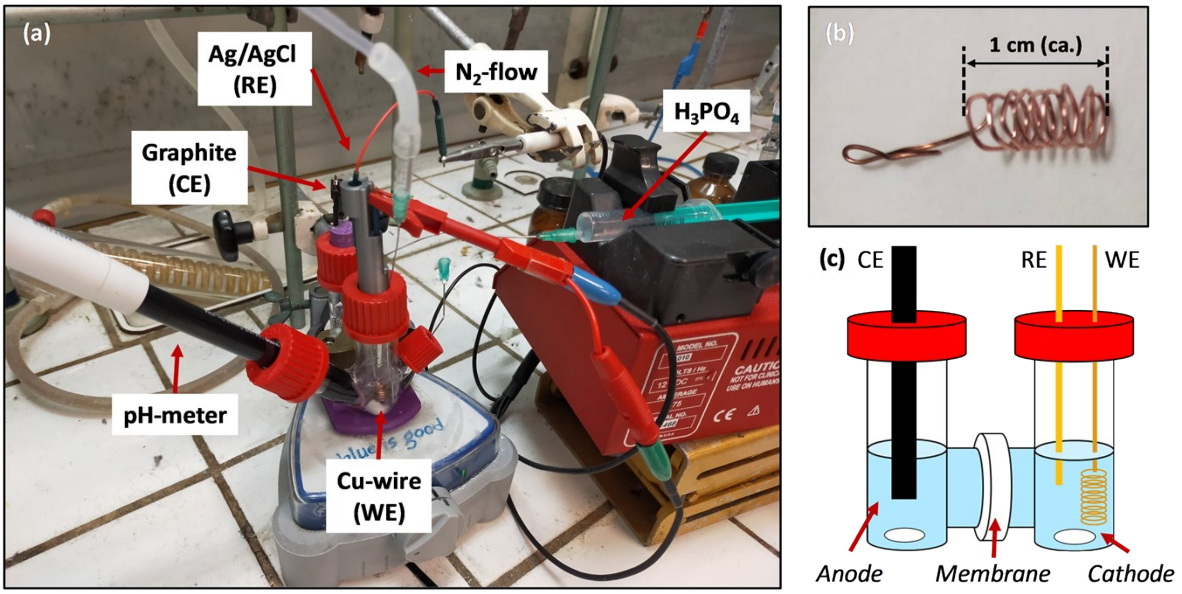


*Figure S2. Picture (a, b) and representation (c) of the electrocatalytic setup and the coiled Cu-wire used as cathode (S.A. = 3.78 cm$^2$) for the FRR experiments.*

*Table S1. Summary of the results of the electrocatalytic reduction of 0.1 M fructose in a 0.1 M $Na_2SO_4$ solution at pH = 6.8 and room temperature (21 °C). Five different potentials were applied in the range -0.5 to -1.0 V vs RHE and the following parameters were measured: pH after 1 h electrolysis ($pH_f$), fructose isomerisation (percentage of initial fructose converted to glucose and mannose), electrochemical conversion (percentage of initial fructose converted to sorbitol and mannitol), sorbitol selectivity (percentage of sorbitol in the sorbitol + mannitol product composition), and Faradaic efficiency (percentage of the e- that were employed to produce sorbitol and mannitol).*

| *E* (V) vs RHE | *Q* (C) | $pH_f$ | Isomerisation (%) | Electrochem. Conv. (%) | Sorbitol selectivity (%) | FE towards FRR (%) |
|---|---|---|---|---|---|---|
| -0.5 | -0.4 | 6.8 | 0.1 | 0.0 | 0 | 0.0 |
| -0.6 | -0.6 | 6.7 | 0.1 | 0.0 | 0 | 0.0 |
| -0.7 | -1.5 | 8.8 | 0.2 | 0.0 | 0 | 1.4 |
| -0.8 | -3.1 | 10.6 | 0.6 | 0.4 | 100 | 16.4 |
| -0.9 | -10.1 | 11.3 | 8.9 | 1.4 | 23 | 19.2 |
| -1.0 | -20.5 | 11.6 | 15.0 | 2.7 | 33 | 17.5 |

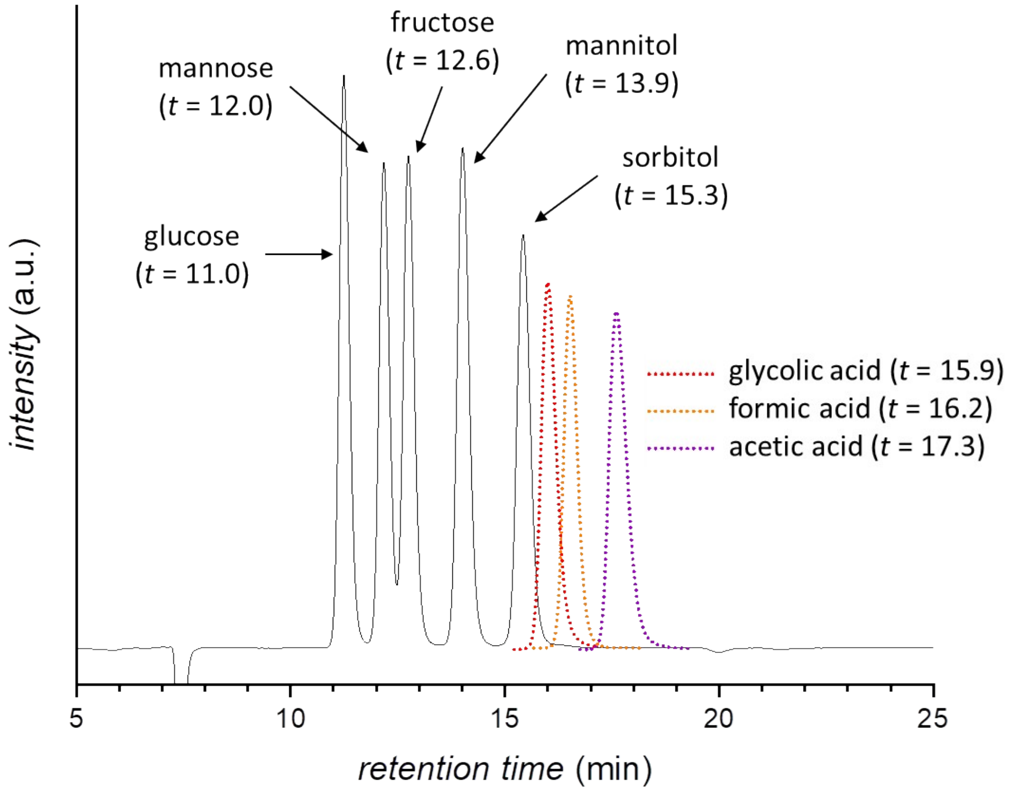


*Figure S3. HPLC chromatogram showing the main species observed during the electrocatalytic reduction of fructose. In solid black, fructose (substrate, t = 12.6 min) and other saccharide species coming from the chemical isomerisation (glucose at t = 11.0 min and mannose at t = 12.0 min) and electrochemical conversion (mannitol at t = 13.9 min and sorbitol t = 15.3 min). In dashed, degradation species: glycolic acid (red, t = 15.9 min), formic acid (orange, t = 16.2 min) and acetic acid (purple, t = 17.3 min).*

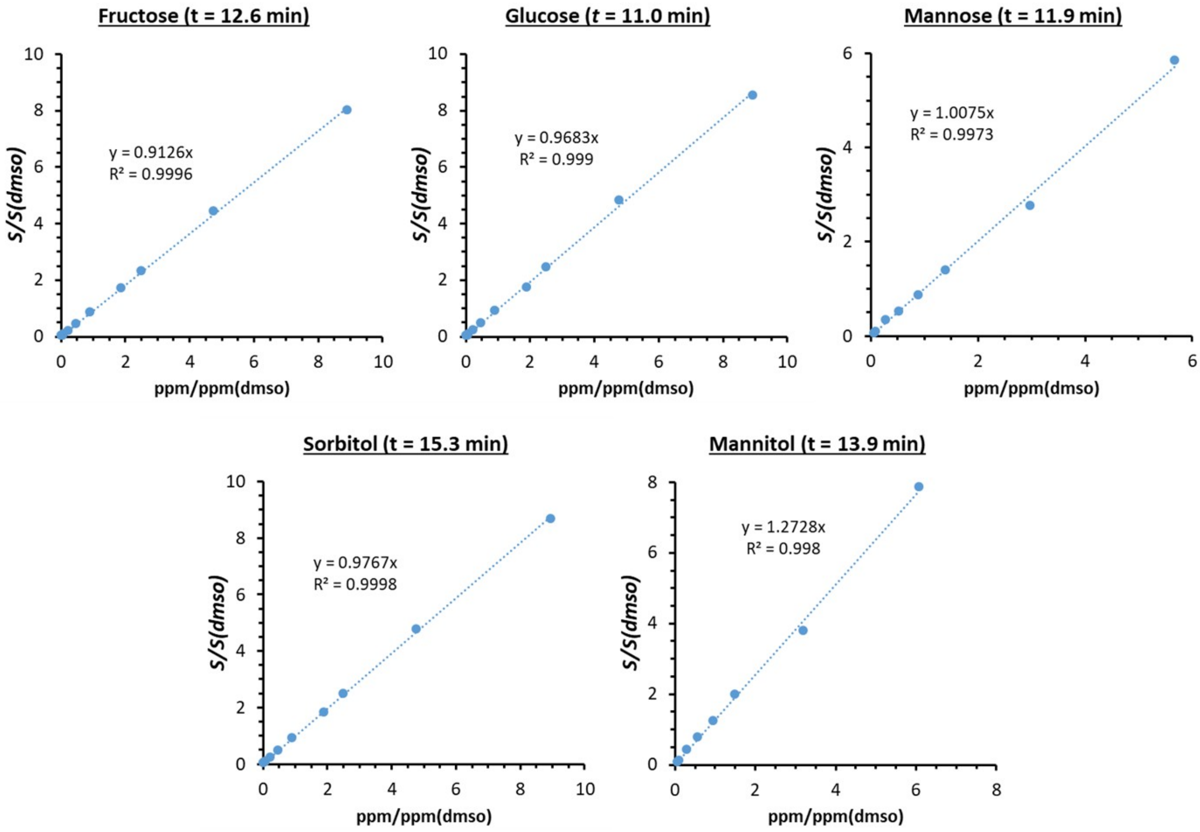


*Figure S4. HPLC calibration curves for fructose and its main chemical (glucose and mannose) and electrochemical (sorbitol and mannitol) conversion products. DMSO was used as internal standard. The plot shows the relationship of the substrate concentration relative to the internal standard (ppm/ppm$_{DMSO}$) with the area of the peak of each molecule relative to the area of the DMSO peak (S/S$_{DMSO}$).*

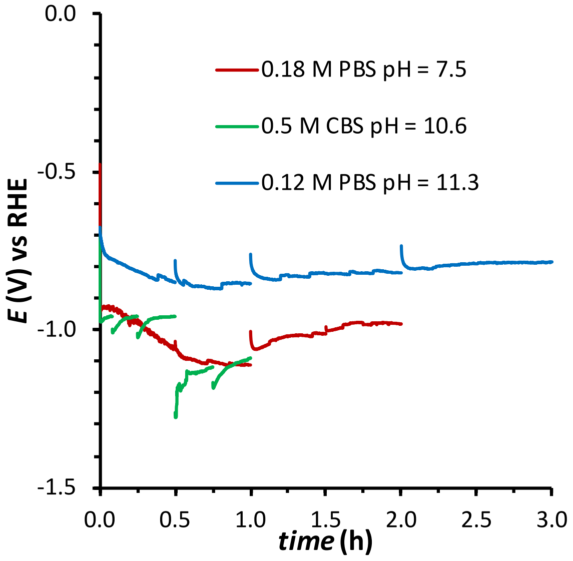


*Figure S5. CP (i = -10 mA or j = 2.65 mA·cm$^{-2}$) curves for a Cu-wire cathode in the presence of 0.1 M fructose solution in 0.18 M PBS pH = 7.5 (red), 0.5 M CBS pH = 10.6 (green) and 0.12 M PBS pH = 11.3 (blue), at room temperature (21 °C).*

*Table S2. Summary of the results of the electrocatalytic reduction of 0.1 M fructose in different electrolytes and pHs at a fixed i = -10 mA (j = 2.65 mA·cm$^{-2}$) with a Cu-wire electrode, at room temperature (21 °C).*

| Electrolyte | *t* (h) | pH | Isomerisation (%) | Electrochem. Conv. (%) | Sorbitol selectivity (%) | FE towards FRR (%) |
|---|---|---|---|---|---|---|
| 0.18 M PBS $pH_{initial}$ = 7.5 | 0.5 | 7.5 | 0.64 | 0.33 | 35 | 3.4 |
| | 1 | 7.8 | 0.19 | 0.92 | 44 | 4.7 |
| 0.5 M CBS $pH_{initial}$ = 10.6 | 0.5 | 10.5 | 0.45 | 1.12 | 43 | 11.6 |
| | 1 | 10.6 | 0.42 | 2.38 | 38 | 12.1 |
| 0.12 M PBS $pH_{initial}$ = 11.3 | 0.5 | 11.1 | 0.12 | 2.25 | 49 | 24.4 |
| | 1 | 11.3 | 0.45 | 3.92 | 43 | 21.1 |
| 0.5 M PBS $pH_{initial}$ = 11.3 | 0.5 | 11.3 | 0.45 | 2.12 | 57 | 22.4 |
| | 1 | 11.4 | 0.48 | 4.13 | 50 | 21.7 |

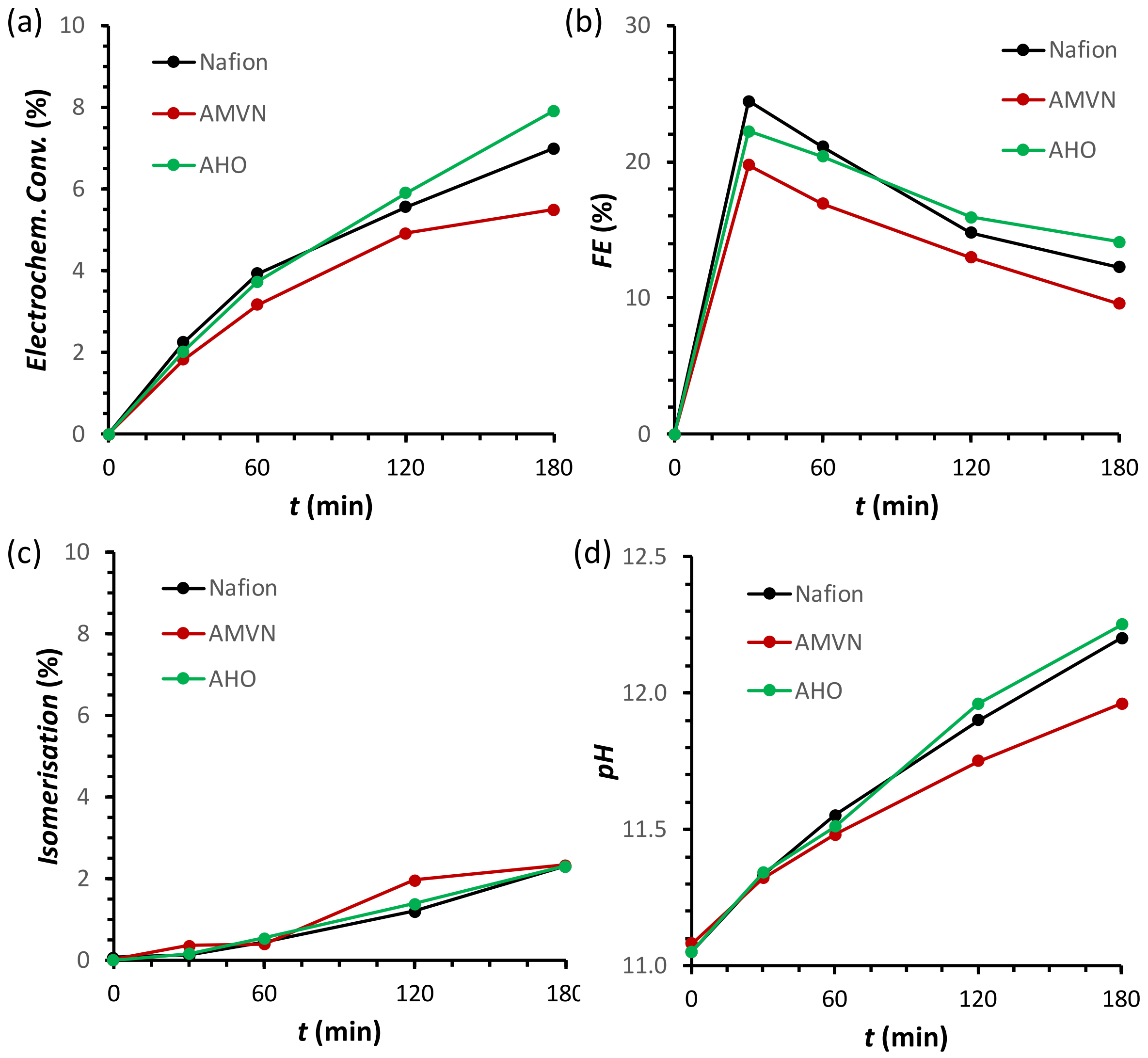


*Figure S6. Chronopotentiometric study of FRR (0.1 M fructose) on a Cu-wire electrode as a function of the membrane: Nafion (black), AMVN (red) and AHO (green), at room temperature (21 °C). The following quantities are reported as a function of the electrolysis time: (a) electrochemical conversion; (b) Faradaic efficiency; (c) isomerisation degree; (d) pH in the cathodic compartment.*

*Table S3. Summary of the results of the electrocatalytic reduction of 0.1 M fructose in a 0.12 M PBS solution at pH = 11.3 with a Cu cathode via CP, at different fixed currents.*

| *i* (mA) | *t* (h) | pH | Isomerisation (%) | Electrochem. Conv. (%) | Sorbitol selectivity (%) | FE towards FRR (%) |
|---|---|---|---|---|---|---|
| -2 | 0.5 | 11.1 | 0.12 | 0.3 | 47 | 17 |
| | 1 | 11.1 | 0.15 | 0.5 | 49 | 13 |
| -5 | 0.5 | 11.2 | 0.13 | 1.5 | 47 | 31 |
| | 1 | 11.3 | 0.27 | 2.2 | 47 | 23 |
| -10 | 0.5 | 11.3 | 0.12 | 2.3 | 49 | 24 |
| | 1 | 11.6 | 0.45 | 3.9 | 43 | 21 |
| -20 | 0.5 | 11.5 | 0.30 | 4.8 | 45 | 25 |
| | 1 | 11.8 | 0.83 | 8.3 | 50 | 21 |

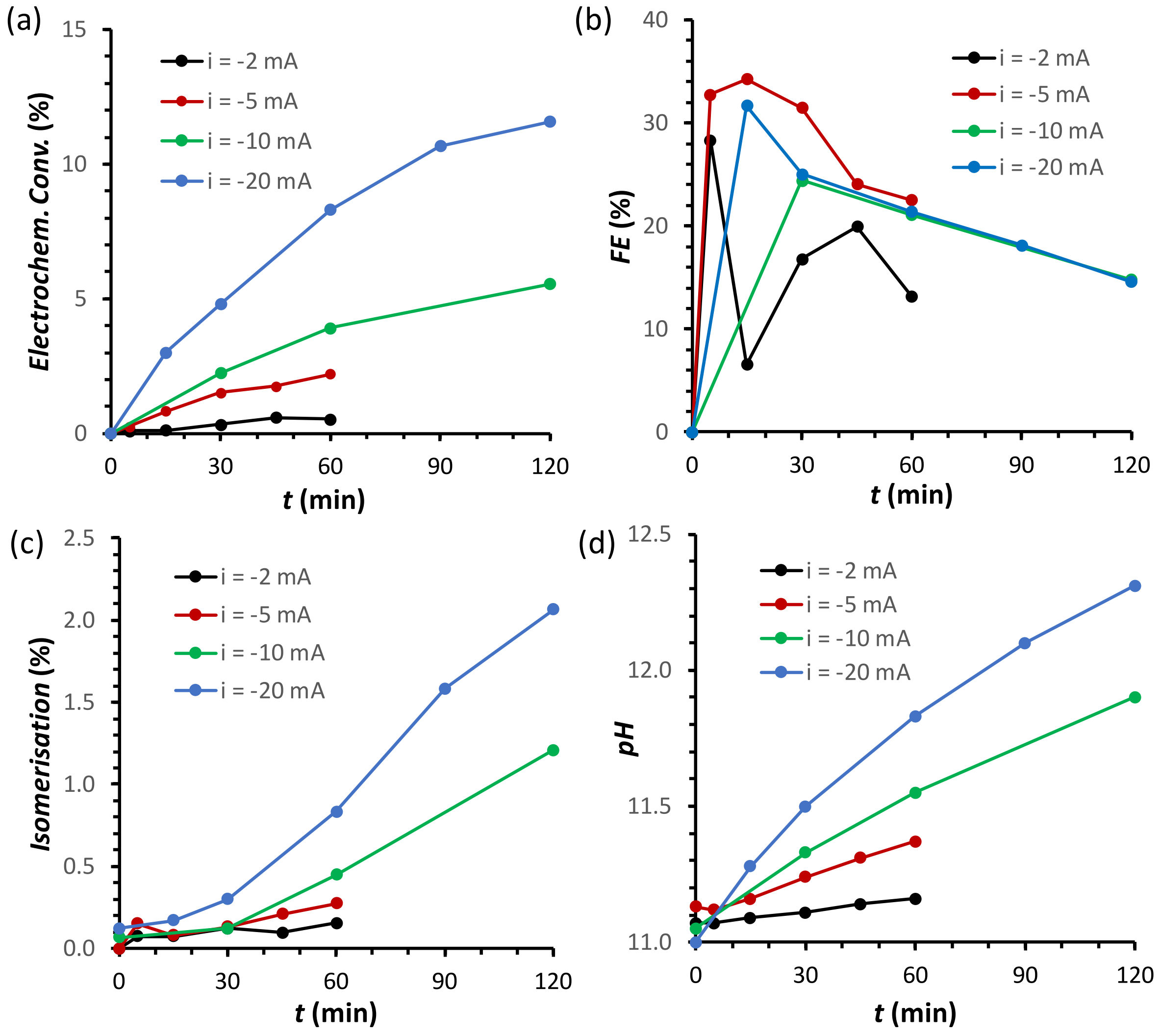


*Figure S7. Chronopotentiometric study of FRR (0.1 M fructose) on a Cu-wire electrode as a function of the current density: -2 mA (black), -5 mA (red), -10 mA (green) and -20 mA (blue), at room temperature (21 °C). The following quantities are reported as a function of the electrolysis time: (a) electrochemical conversion; (b) Faradaic efficiency; (c) isomerisation degree; (d) pH in the cathodic compartment. Note: in the experiments at i = - 2 mA (as well as the data points at 15 min for the other tests), the concentration of the products of the electrochemical and chemical reactions were very low, causing a higher degree of uncertainty in their quantification and thus probably leading to the fluctuations observed in the FE values at this current.*

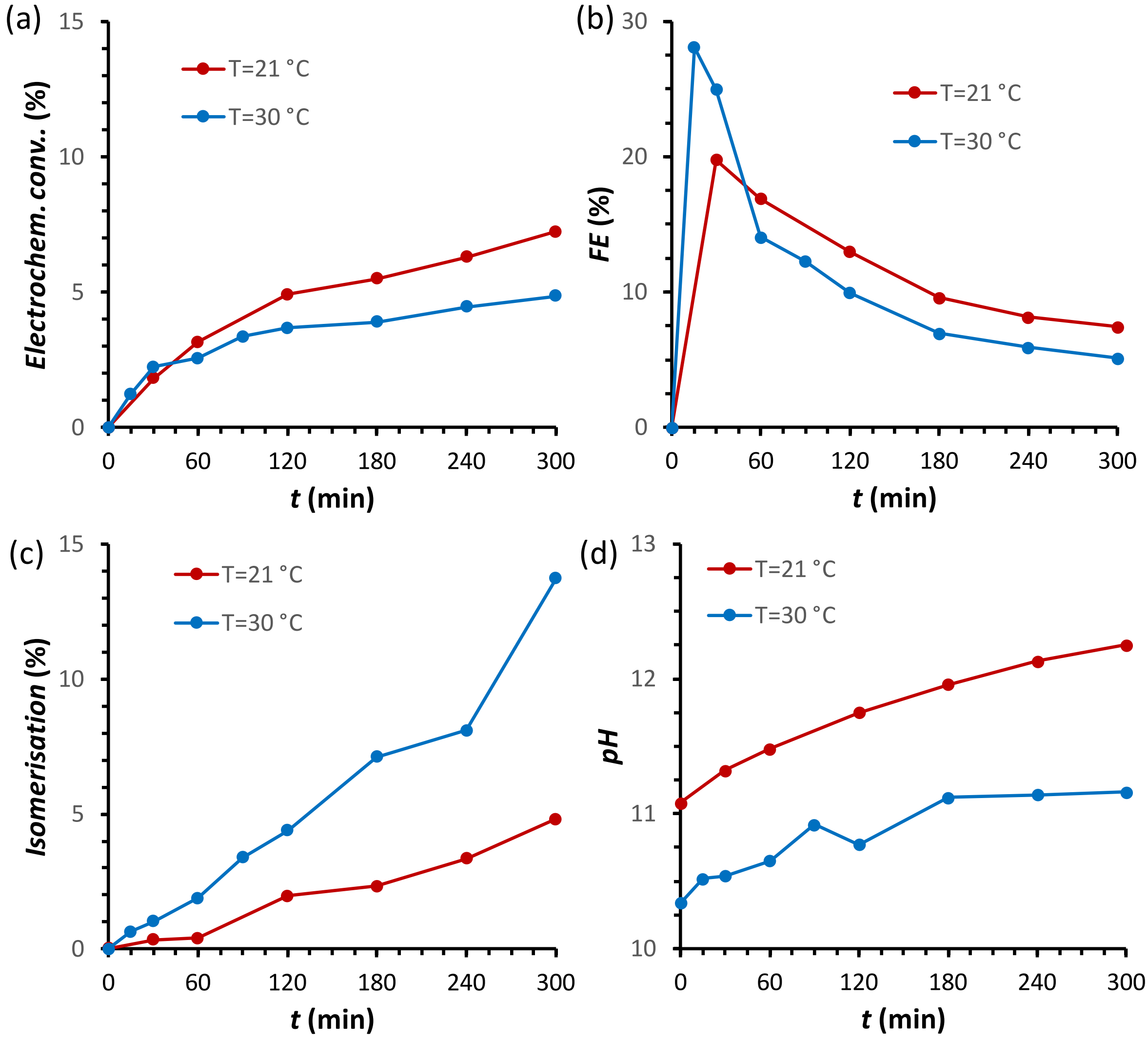


*Figure S8. Chronopotentiometric study of FRR (0.1 M fructose) on a Cu-wire electrode at i =* -10 mA (*j* = 2.65 mA·cm$^{-2}$) *as a function of the temperature:* r.t. (red, 21 °C) and at T = 30 °C (blue) *The following quantities are reported as a function of the electrolysis time: (a) electrochemical conversion; (b) Faradaic efficiency; (c) isomerisation degree; (d) pH in the cathodic compartment.*

*Table S4. Summary of the results of the electrocatalytic reduction of 0.1 M fructose in a 0.12 M PBS solution at pH = 11.3 with a Cu-wire electrode at a fixed i = -20 mA, in the absence (orange) and presence (grey) of a titration pump operated with 0.5 M $H_3PO_4$ (10 µL·min$^{-1}$) to keep the pH constant. Room temperature (21 °C).*

| ***$H_3PO_4$ addittion*** | ***t* (h)** | **pH** | **Isomerisation (%)** | **Electrochem. Conv. (%)** | **Sorbitol Selectivity (%)** | **FE towards FRR (%)** |
|---|---|---|---|---|---|---|
| None | 1 | 11.5 | 0.30 | 4.8 | 45 | 25 |
| | 2 | 11.8 | 0.83 | 8.3 | 50 | 21 |
| | 3 | 12.6 | 4.29 | 15.9 | 48 | 13 |
| 10 µL·min$^{-1}$ | 1 | 11.0 | 0.14 | 6.1 | 47 | 32 |
| | 2 | 11.1 | 0.24 | 8.6 | 47 | 23 |
| | 3 | 10.9 | 1.20 | 18.5 | 47 | 16 |

*Table S5. Summary of the results of the electrocatalytic reduction of 0.1 M fructose in a 0.12 M PBS solution at pH = 11.3 with a Cu-wire electrode at a fixed i = -20 mA, when using no pulse (grey) or the following oxidative pulses: 0.47 V (maroon), 0.87 V (green) and 1.37 V (blue). The pulses were applied during a CP experiment every 5 min and for 5 s, and the electrolysis was immediately restarted. A titration pump was used to keep the pH constant by providing 10 µL·min$^{-1}$ of 0.5 M $H_3PO_4$. Room temperature (21 °C).*

| ***Pulses*** | ***t* (h)** | **pH** | **Isomerisation (%)** | **Electrochem. Conv. (%)** | **Sorbitol Selectivity (%)** | **FE towards FRR (%)** |
|---|---|---|---|---|---|---|
| None | 0.5 | 11.0 | 0.14 | 6.1 | 47 | 32 |
| | 1 | 11.1 | 0.24 | 8.6 | 47 | 23 |
| | 2 | 11.3 | 0.62 | 14.9 | 44 | 20 |
| 0.47 V | 0.5 | 11.1 | 0.09 | 5.8 | 48 | 33 |
| | 1 | 11.1 | 0.21 | 11.2 | 46 | 32 |
| | 2 | 11.1 | 0.38 | 17.5 | 46 | 25 |
| 0.87 V | 0.5 | 11.2 | 0.11 | 7.4 | 44 | 41 |
| | 1 | 11.2 | 0.11 | 7.4 | 44 | 41 |
| | 2 | 11.0 | 0.21 | 15.4 | 51 | 42 |
| 1.37 V | 0.5 | 11.3 | 0.14 | 8.2 | 47 | 45 |
| | 1 | 11.3 | 0.14 | 8.2 | 47 | 45 |
| | 2 | 11.2 | 0.53 | 29.7 | 46 | 41 |

## S1.1 Cu-leaching from the electrode

In some publications, the leaching of Cu has been detected under similar reaction conditions to those used in this work via elemental analyses of the electrolyte solution.[2,3] For instance, it was found that in 0.05 M KOH (pH = 12.7), Cu dissolves at around 0.4 V vs SHE (1.1 V vs RHE).[2] The leaching was reported to be pH-dependent, with pH = 8-10 being the optimal range (low amount or no Cu detected in solution). If the pH was increased (0.1 M KOH, pH = 13), the oxidation was boosted and thus the leaching started at lower oxidative potentials (0.67 V vs RHE).[3] The leaching of Cu in our system was studied by Inductively-Coupled Plasma Optical Emission Spectroscopy (ICP-OES). The data were compared to the ICP-OES results of Milli-Q water, PBS solution and PBS solution in the presence of 0.1 M fructose (solution 1: s1). The just-polished Cu electrode was dipped for 5 min in s1, and the ICP-OES result (Table S6, entry 4) proves that the leaching under these conditions, if any, was below the detection limit of the equipment (< 0.1 ppm). This result suggests that, although there was a superficial oxidation/passivation at the OCV when dipping the electrode in the reaction solution, the leaching of Cu was negligible at this pH and in the exposed timeframe.

*Table S6. ICP-OES results of solutions at different reaction conditions. AVG = average of 2 measurements; STD = standard deviation of 2 measurements.*

| Entry | Sample | Expected concentration (ppm) | AVG | STD | nmol | µM |
|---|---|---|---|---|---|---|
| 1 | Milli-Q water | 0 | < 0.1 | | < 15.7 | < 1.6 |
| 2 | *I* = 0.5 M PBS (s0) | 0 | < 0.1 | | < 15.7 | < 1.6 |
| 3 | s0 + 0.1 M fructose (s1) | 0 | < 0.1 | | < 15.7 | < 1.6 |
| 4 | Cu immersion into s1 (5 min) | < 10 | < 0.1 | | < 15.7 | < 1.6 |
| 5 | 5 min reduction at *i* = -20 mA in s1 | < 10 | 0.10 | 0.00 | 15.7 | 1.6 |
| 6 | 5 min reduction at *i* = -20 mA in s1 + 5 s oxidation at *E* = 1.37 V | < 10 | 0.40 | 0.00 | 62.9 | 6.3 |
| 7 | 5 min reduction *i* = -20 mA s1 + 5 s oxidation *E* = 1.37 V + 5min reduction *i* = -20 mA | < 10 | 0.40 | 0.00 | 62.9 | 6.3 |
| 8 | 20 min *i* = -20 mA | < 10 | 0.10 | 0.00 | 15.7 | 1.6 |

Then, the possible Cu leaching at the different reaction conditions was studied. A series of samples were independently prepared and analysed by ICP-OES (Table S6) at the following stages: after 5 min electrolysis at *i* = -20 mA (entry 5); after 5 min electrolysis at *i* = -20 mA and 5 s oxidative pulse at 1.37 V (entry 6); after 5 min electrolysis at *i* = -20 mA, 5 s pulse at 1.37 V and 5 more min electrolysis at *i* = -20 mA (entry 7); and after 20 min electrolysis at *i* = -20 mA without any oxidative pulse (entry 8). According to the concentration values, it can be concluded that during the electrolysis (entries 5-8) there is a small degree of leaching of Cu, which is not observed by only immersing the electrode in the PBS solution containing fructose. The leaching increases ca. 4 times when the 5 s oxidative pulse is applied after the 20 min electrolysis (entry 6). In the case in which another 5 min FRR electrolysis was performed after the oxidative pulse (entry 7), similar ICP-OES results were obtained. According to the Pourbaix diagram (Figure S14), at 1.37 V vs RHE $Cu_2O$ gets oxidised to CuO, which has a low solubility of $2{\cdot}10^{-7}$ M and could thus precipitate in the reaction vessel.[4] Cu dissolution has been observed by other authors in 0.05 M KOH starting at 1.37 V and more intensely at 0.78 V (RHE) when being oxidised from Cu(I) to Cu(II).[2]

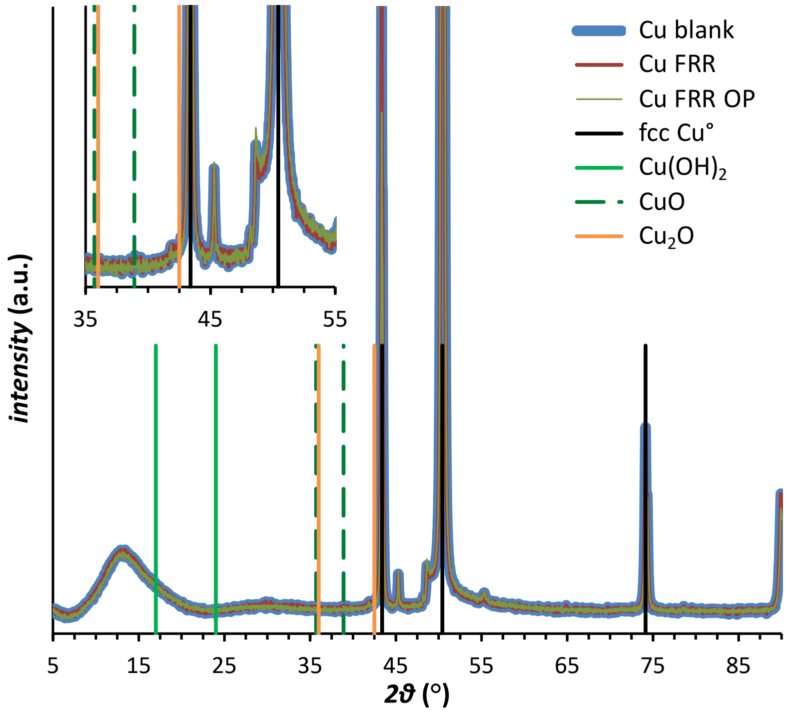


*Figure S9. X-ray diffraction (XRD) analysis of different electrodes: Cu blank (pristine Cu electrode), Cu FRR (electrode after 10 h electrolysis at i = -20 mA·cm-2), and Cu FRR OP (electrode after 10 h electrolysis at i = -20 mA·cm-2 including 5 s oxidative pulses at 1.37 V every 5 min). Conditions: 0.1 M fructose, AMVN membrane as separator, constant pH titration with 10 µL·min-1 of 0.5 M $H_3PO_4$, room temperature (21 °C).*

XRD analyses (Figure S9) show no change in the crystallographic structure during the electrochemical tests. Diffraction peaks appear at 2θ values of 43.40°, 50.43°, 74.14°, in good agreement with the standard pattern for pure face-centred cubic phase of $Cu^0$ (JCPDS No. 040836).[5] No peaks at ~36° or 42.5° corresponding to (111) and (200) of $Cu_2O$ (respectively) were observed,[6] or at 35.7° and 38.9° from (002) and (111) due to monoclinic CuO,[7,8] or ~17° and ~24° from (020) and (021) from the orthorhombic $Cu(OH)_2$ crystal system.[9] The absence of these peaks proves that the passivation of the Cu platedid not result in the formation of crystalline species and thus only an amorphous layer is formed.

## S1.2 XPS analysis

While carrying out the XPS measurements, we noticed that the Cu plate was covered with a transparent and thin polymeric coating, as confirmed by the survey spectra (Figure S10a), which contains C, N and Si (C 1s at 285 eV, N 1s 400 eV and Si 2p at 102 eV). This coating could be largely removed by polishing the electrode (compare the data of the pristine and polished Cu plate in Figure S10,b-d).

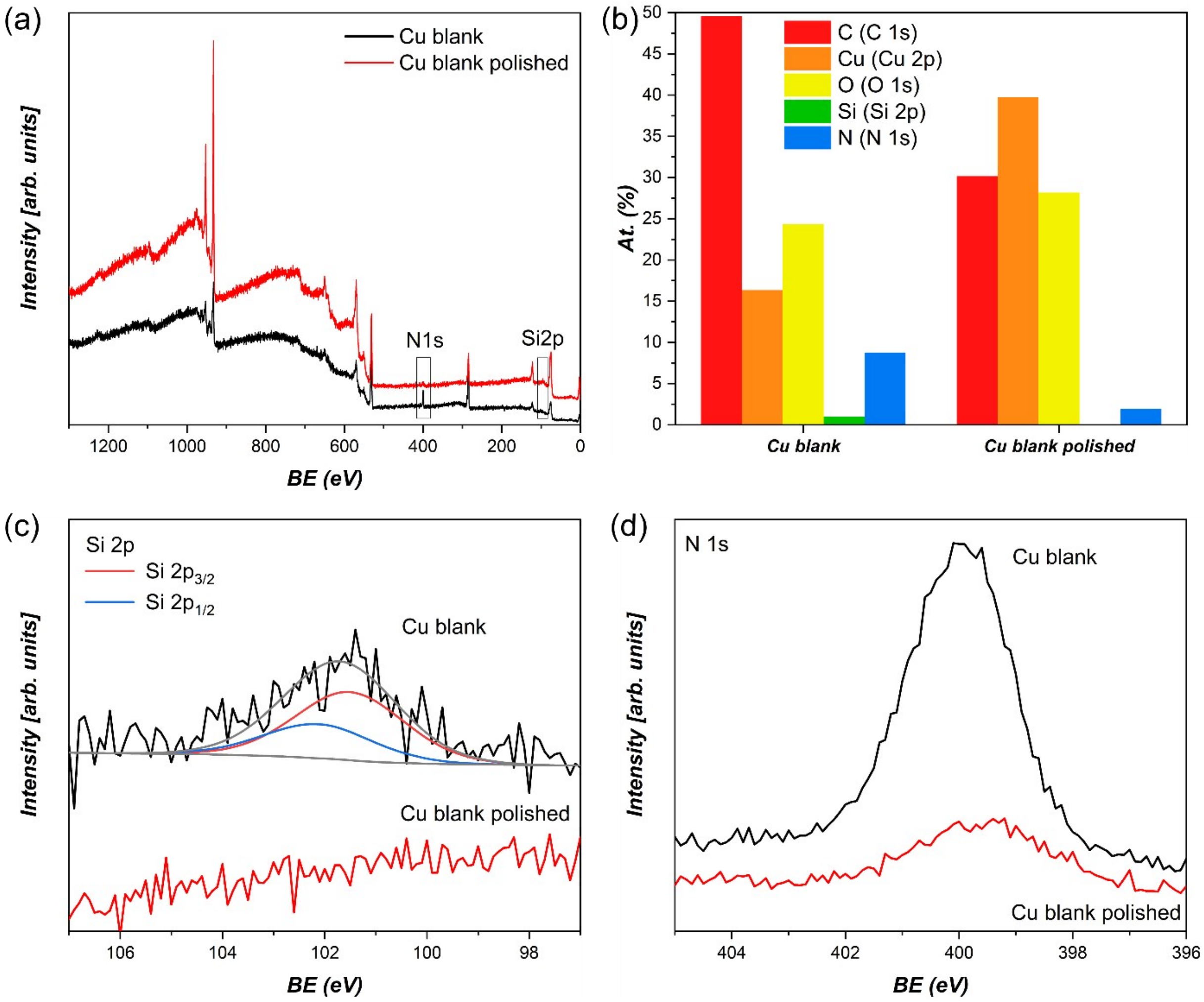


*Figure S10. Comparison between the XPS data of the pristine Cu plate electrode (Cu blank) and of the polished one (Cu blank polished): (a) survey spectra, (b) relative atomic content, (c) Si 2p core level region with relative fit and (d) N 1s core level region.*

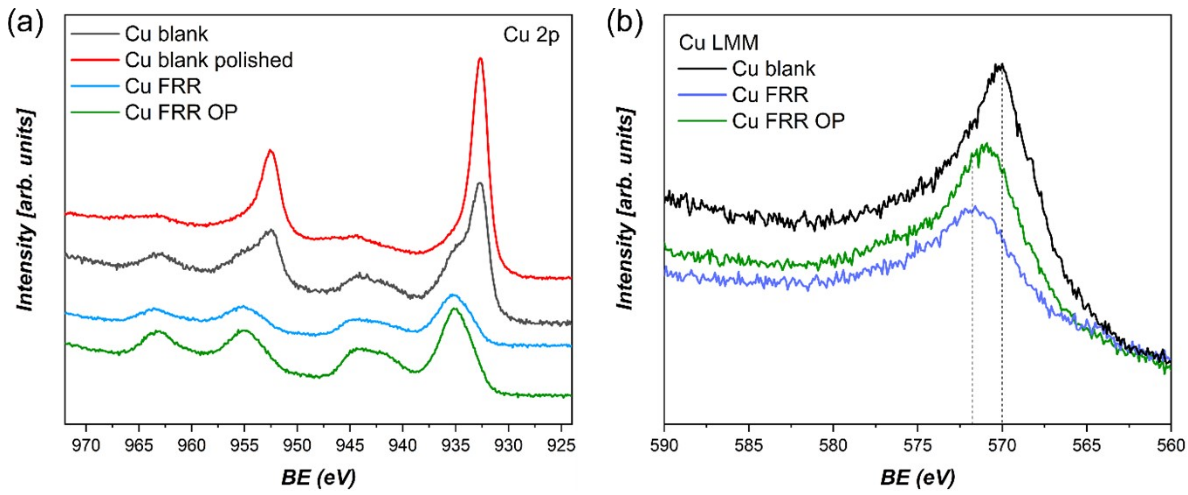


*Figure S11. XPS spectra of the Cu 2p core level region and the Cu LMM Auger peaks of Cu blank, Cu blank polished, Cu FRR and Cu FRR OP and corresponding fits.*

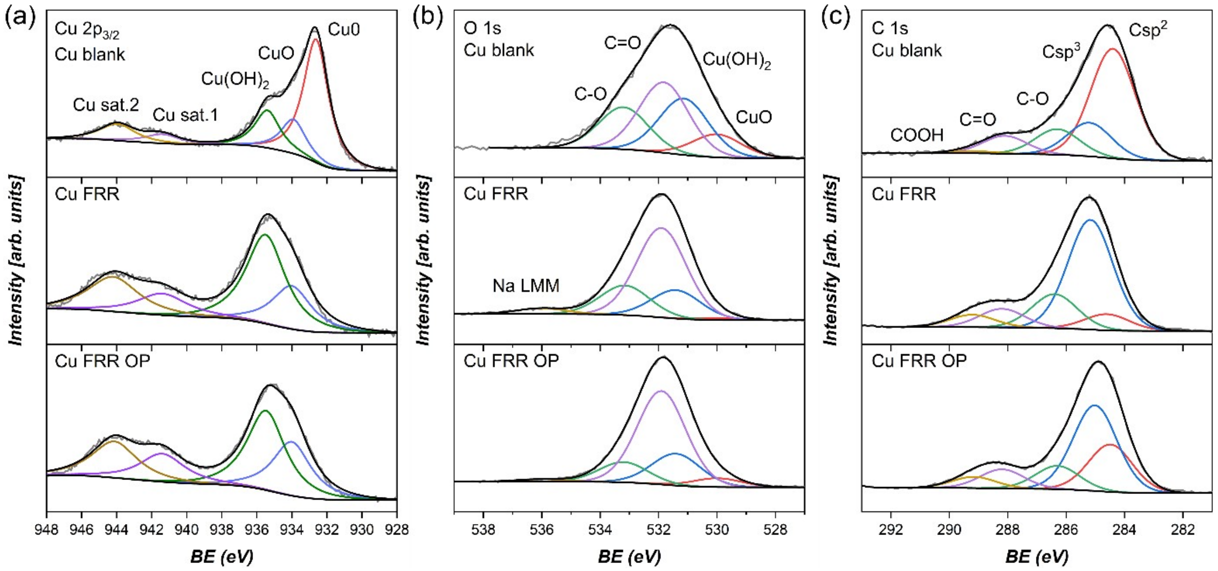


*Figure S12. XPS spectra of the Cu 2p, O 1s and C 1s core level regions of Cu blank, Cu FRR and Cu FRR OP, and their fits.*

*Table S7. XPS core level peak assignment.*

| Core level | Assignment | BE (eV) | FWHM (eV) |
|---|---|---|---|
| C 1s | $Csp^2$ | 284.5 ± 0.1 | 1.8 |
| | $Csp^3$ | 285.1 ± 0.1 | 1.8 |
| | C-O | 286.3 ± 0.1 | 1.8 |
| | C=O | 288.1 ± 0.1 | 1.8 |
| | COOH | 289.2 ± 0.1 | 1.8 |
| O 1s | CuO | 529.9 ± 0.1 | 2.0 |
| | $Cu(OH)_2$ | 531.2 ± 0.1 | 2.0 |
| | C=O | 533.1 ± 0.1 | 2.0 |
| | C-O | 531.8 ± 0.1 | 2.0 |
| | Na LMM | 536.0 ± 0.1 | 2.0 |
| Cu $2p_{3/2}$ | $Cu^0$ | 932.6 ± 0.1 | 1.7 |
| | CuO | 933.9 ± 0.1 | 1.7 |
| | $Cu(OH)_2$ | 935.4 ± 0.1 | 2.4 |

**S2. The chemistry of saccharides**

Fructose, glucose and other saccharides can be present in several configurations (tautomers) depending on the conditions at which they are exposed.[10] On the one hand, glucose is found in solid state mainly as a pyranose ring (6-member ring), either in α- or β- configurations. When present in an aqueous neutral solution, the aforementioned α/β tautomers mutarotate until reaching an equilibrium (64 % β, [α] = 52.7°).[10] While in neutral conditions this equilibrium can take more than 3h to be reached,[11] in acidic medium (0.1 M $HClO_4$) the equilibrium is reached faster, and in alkaline conditions (0.1 M NaOH) it is almost instantaneous.[12] During this process, the linear aldehydo form of glucose is present (in very small amounts, 0.024 % at r.t.),[10] and this is essential as this is the electroactive form that gets reduced via electrocatalysis.[10,13] In fact, the mutarotation of the cyclic form to the linear one can become the rate-determining step of the electrocatalytic reduction, which in such case is pH-dependent and potential-independent.[13] Apart from these configurations, glucose can also be found in the furanose forms (α/β) in aqueous solution, although these tautomers are rather unstable.

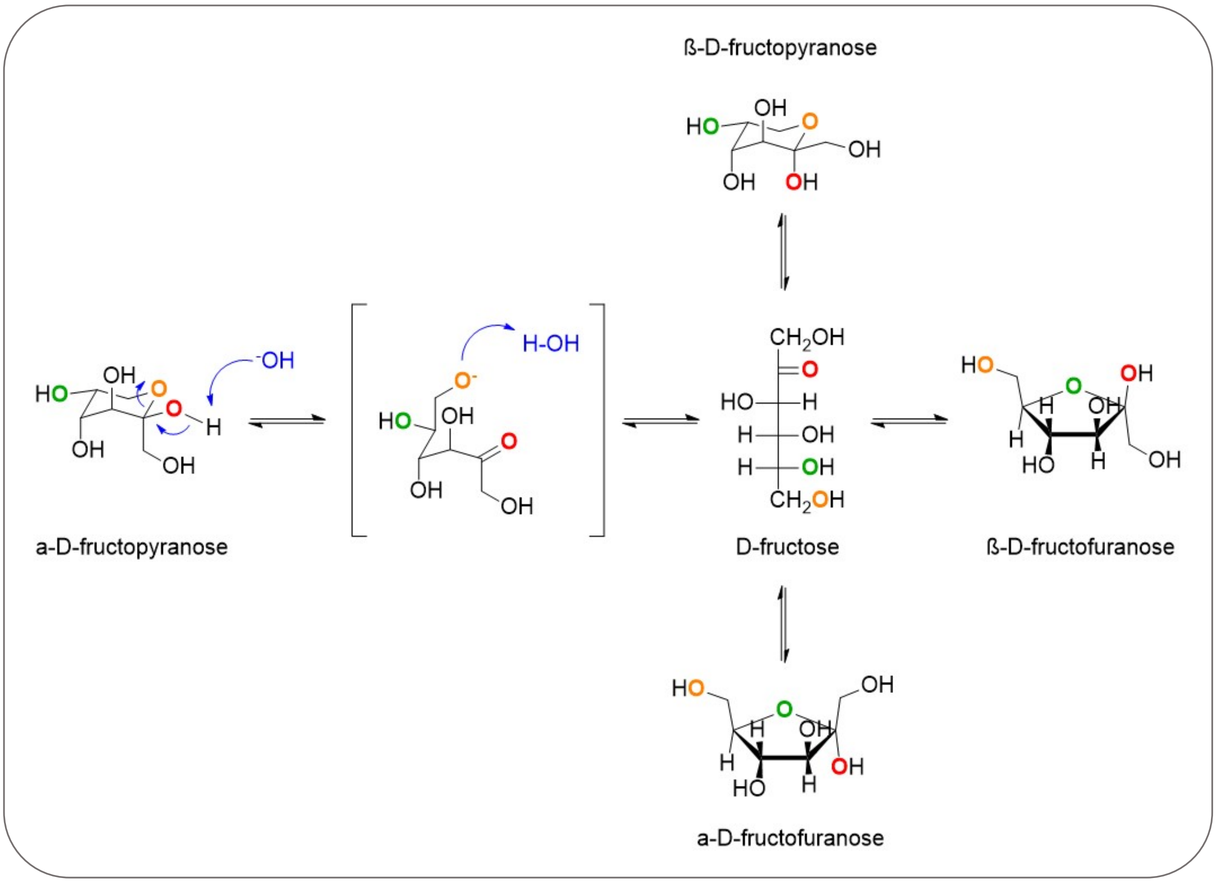


*Scheme S1. Representation of the mutarotation of D-fructose in aqueous conditions. In blue, representation of the possible reaction mechanism in alkaline medium. In red, O-atom corresponding to the ketone group; in orange, $C_6$-OH group involved in the cyclisation for the formation of D-fructopyranose rings; in green, $C_5$-OH group involved in the cyclisation for the formation of D-fructofuranose rings.*

Fructose production from base-catalysed glucose isomerisation is a well-known process that is already implemented on an industrial scale.[14] Glucose conversion to fructose increases with the solution alkalinity, reaching a steady maximum in the pH = 10.7-11.3 range, and decreases again at pH > 11.5 due to degradation reactions.[15] Fructose is commercially available in its crystalline form as D-(-)-fructopyranose, containing both the α and β tautomers.[16,17] Its mutarotation (Scheme S1) has been studied in aqueous solution at pH = 4.4 in the 10-55 °C temperature range, showing a higher

mutarotation rate than glucose. The authors found that the β-fructopyranose tautomer is the most abundant one (between 76 mol% at 10 °C and 66 mol% at 55 °C) in all the temperature range. With the increase on the temperature, the equilibrium slightly shifts towards the formation of the 5-membered rings β-fructofuranose (from 19 to 26 %) and α-fructofuranose (from 4 to 7 %), with the permanent presence of the linear keto form (0.2-0.4 %).[16] In $D_2O$, the acyclic keto form of fructose can account up to 0.5-2 %,[17,18] 1-2 orders of magnitude more concentrated than the aldehydo one from glucose (0.024 %).[10] The faster mutarotation and higher concentration of the acyclic (linear) form make fructose a more redox-reactive substrate than glucose. Considering that the mutarotation of saccharides is enhanced under alkaline conditions, the electrocatalytic reduction of this type of substrates is driven by near-surface pH changes:[13,19] when a reductive potential is applied, the pH at the vicinity of the cathode increases (in contrast to the bulk pH) due to the consumption of $H^+$ or generation of $OH^-$ resulting from the HER, catalysing the aforementioned glucose/fructose mutarotation.

As soon as fructose is solubilised in a 0.12 M PBS electrolyte solution, a pH decrease is observed, presumably due to the ring-opening mechanism (Scheme S1) starting from a deprotonated form of fructose (see Table S8 for the initial pH of the electrolyte and the pH after addition of fructose).[15] The occurrence of this decrease depends from several reaction parameters. For instance, no pH change is observed at neutral pH upon fructose addition (Table S8, entries 1 and 2), while in the 10.5-13 range, the more alkaline the pH is, the larger pH shift is observed upon addition of fructose, as a result of a higher chemical conversion rate (entries 3-12). Moreover, in the case of buffer solutions, the higher the concentration of the electrolyte, the smaller the pH change due to the buffering effect (entries 6-8). Finally, the higher the concentration of substrate, the higher the pH decrease due to a higher number of substrate molecules getting deprotonated (entries 4, 9 and 10). When solubilising glucose instead of fructose (entry 12), a smaller pH decrease is observed, presumably due to the earlier mentioned higher faster mutarotation rate between fructose tautomers compared to glucoses tautomers. Reported kinetic studies with isotope effect on the glucose molecule suggest that the deprotonation of the Cα to the carbonyl group (C1 in the case of fructose, C2 in the case of glucose) plays a key role in the mutarotation and further isomerisation process.[15]

*Table S8. pH variation upon addition of fructose (and glucose) in experiments carried out in different electrolytes, pH, concentrations, temperature and organic substrate. The pH was measured before any electrochemical experiment was run, at room temperature (21 °C, unless otherwise stated).*

| Entry | [electrolyte] | $[OH^-]$ (M) | [fruct] (M) | $pH_{initial}$ | $pH_{fruc}$ | ΔpH |
|---|---|---|---|---|---|---|
| 1 | ***[$Na_2SO_4$] = 0.1 M*** | $6.3\cdot10^{-8}$ | 0.10 | 6.80 | 6.80 | 0.00 |
| 2 | ***[PBS] = 0.18 M*** | $2.9\cdot10^{-7}$ | 0.09 | 7.46 | 7.46 | 0.00 |
| 3 | ***[CBS] = 0.5 M*** | $3.8\cdot10^{-4}$ | 0.10 | 10.58 | 10.51 | 0.07 |
| 4 | ***[PBS] = 0.12 M*** | $1.9\cdot10^{-3}$ | 0.10 | 11.28 | 11.12 | 0.16 |
| 5 | ***[NaOH] = 0.1 M*** | $1.3\cdot10^{-1}$ | 0.10 | 13.10 | 12.77 | 0.33 |
| 6 | ***[PBS] = 0.013 M*** | $1.8\cdot10^{-3}$ | 0.10 | 11.25 | 10.57 | 0.68 |
| 7 | ***[PBS] = 0.026 M*** | $2.2\cdot10^{-3}$ | 0.10 | 11.34 | 10.85 | 0.49 |
| 8 | ***[PBS] = 0.5 M*** | $2.0\cdot10^{-3}$ | 0.10 | 11.30 | 11.13 | 0.17 |
| 9 | ***[PBS] = 0.12 M*** | $2.0\cdot10^{-3}$ | 0.05 | 11.30 | 11.17 | 0.13 |
| 10 | ***[PBS] = 0.12 M*** | $1.2\cdot10^{-3}$ | 0.48 | 11.07 | 10.69 | 0.38 |
| 11 | ***[PBS] = 0.12 M (30 °C)*** | 4.0E-04 | 0.10 | 10.60 | 10.34 | 0.26 |
| 12 | ***[PBS] = 0.12 M*** | 2.0E-03 | [gluc] = 0.10 M | 11.30 | 11.20 | 0.10 |

## S3. Study of the chemical conversion of fructose

Before starting any long-period electrochemical study, it is essential to understand what happens to our substrate molecule under the reaction conditions and time ($t$ = 1-10 hours), in the absence of an applied potential. In aqueous solution, fructose (as well as other monosaccharides) mutarotates to its linear form and to other cyclic forms (see Section S2).[16,17] At the same time, fructose can isomerise to glucose and mannose (see Scheme 1 in the manuscript). Both homogenous reactions (mutarotation and isomerisation) are boosted by the presence of $OH^-$ in solution. In fact, the linear form is required for both processes to take place, making their competition unavoidable.

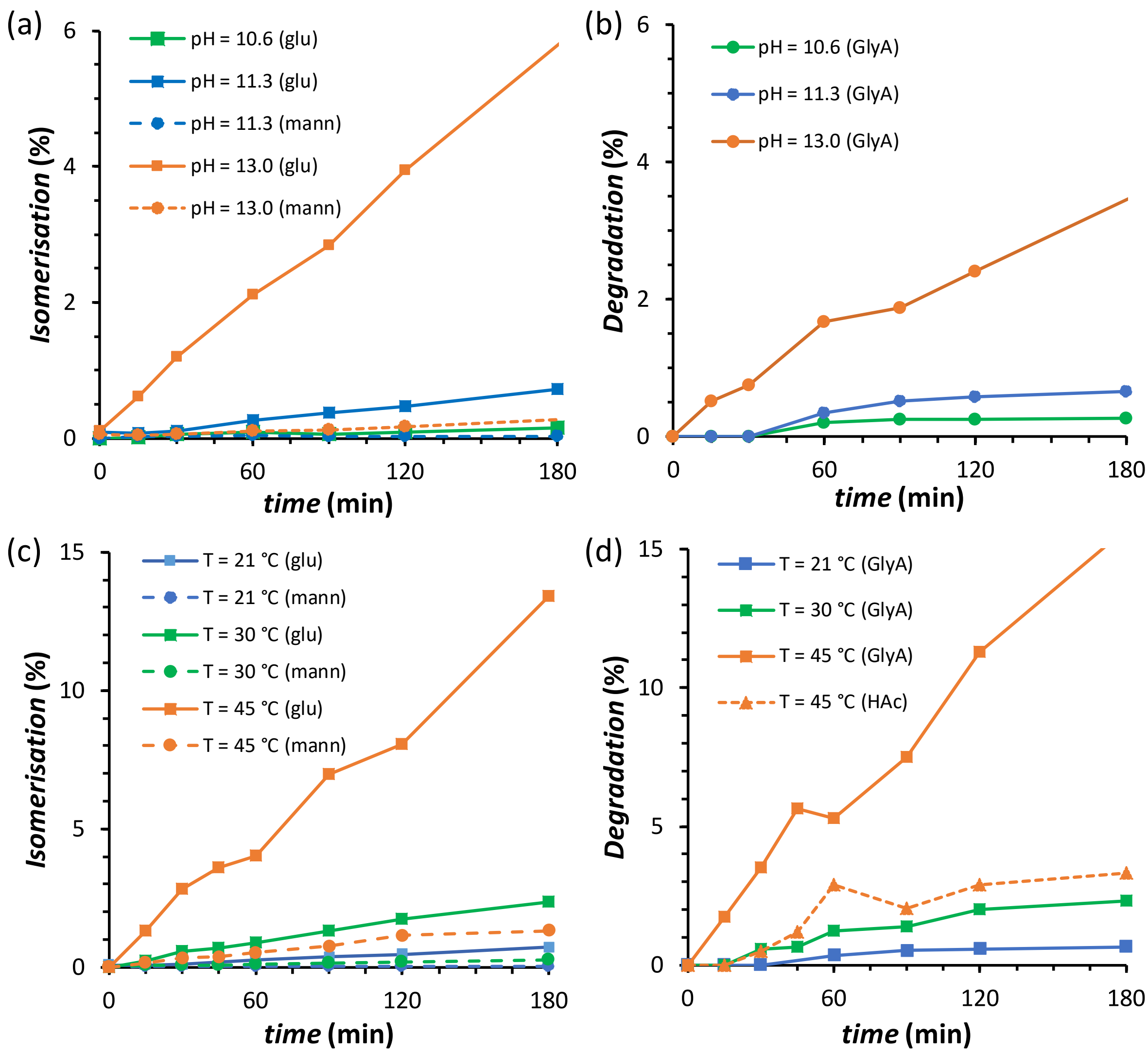


*Figure S13. Fructose chemical conversion at different reaction conditions: pH/electrolyte-dependent isomerisation (a) and degradation (b); temperature-dependent isomerisation (c) and degradation (d). The experiments were performed in an $O_2$-free solution and in a 0.12 M PBS solution (unless otherwise stated). Conditions: 0.1 M fructose, room temperature (21 °C).*

Figure S13 shows the different chemical conversion results (isomerisation and degradation) as a function of the electrolyte, pH and temperature. At pH = 7.5 and room temperature, no chemical conversions were observed within the first 3 hours, and thus the results were not plotted in the graph. Under mild alkaline pH (> 10.5), both isomerisation and degradation start taking place, drastically increasing at pH = 13, thus confirming the influence of the pH on these reactions. The influence of

temperature was studied at pH = 11.3 at 30 and 45 °C. As it can be observed in Figure S7c and S7d, increasing the temperature also induces chemical conversion reactions, especially at 45 °C. Considering that the chemical conversion rate is constant under the reaction conditions, increasing the electrochemical conversion rate (e.g. by increasing the current density) would allow to achieve higher electrochemical conversion in shorter periods of time, thus increasing the ratio between electrochemical and chemical conversion products. It must be mentioned that increasing the temperature to 30 °C resulted in a shift of the $HPO_4^{2-} \rightleftharpoons PO_4^{3-}$ buffer equilibrium towards the most protonated species, thus decreasing the pH of the system down to 10.6.

**S4. Redox properties of the Cu catalyst**

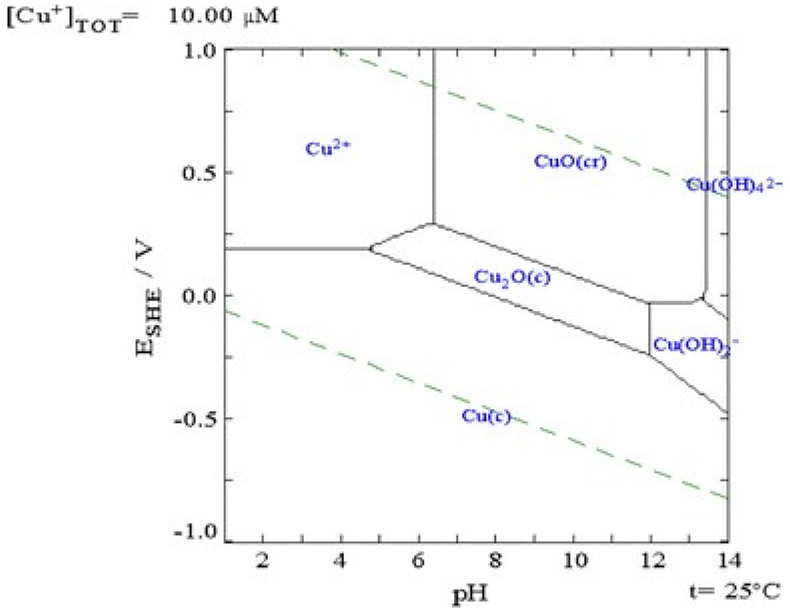


*Figure S14. Pourbaix diagram of Cu, reproduced with permission from reference 20.*

The behaviour of the Cu-wire electrode was studied by means of electrochemical techniques in the absence of fructose. A series of tests were operated in 0.12 M PBS (*I* = 0.5 M, *I*: ionic strength) at pH = 11.3 and room temperature (r.t. 21 °C), in the absence and presence of 0.1 M fructose.

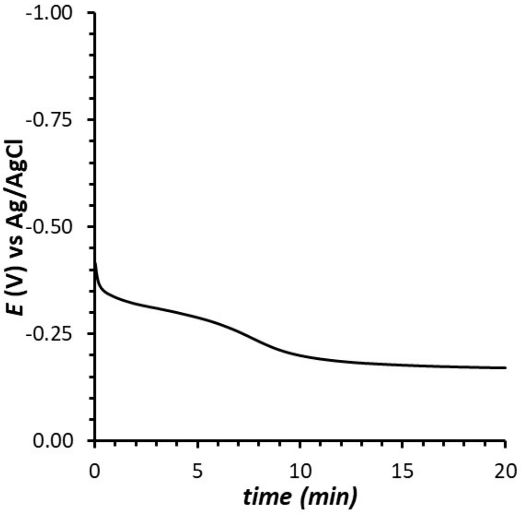


*Figure S15. OCV of a Cu wire electrode (S = 3.78 $cm^2$) in a 0.12 M PBS solution with a graphite rod CE.*

The first feature that we observed before any potential was applied is the measured open circuit potential (OCP or OCV) when the Cu-wire is connected in the presence of the reference electrode (RE, Ag/Ag/Cl): the OCV plot displays a continuous potential decrease towards oxidative potentials (Figure S15). This phenomenon happens as a result of the reaction conditions: at pH = 11.3 and room temperature, Cu corrodes to $Cu^{I}$ forming $H_2$ as by-product, a phenomenon that was already described in a 0.05 M KOH solution,[2] and which can be deduced by looking at the Pourbaix diagram (Figure S14). In fact, it is well known in the scientific community that Cu passivates under weak acidic or alkaline conditions.[21]

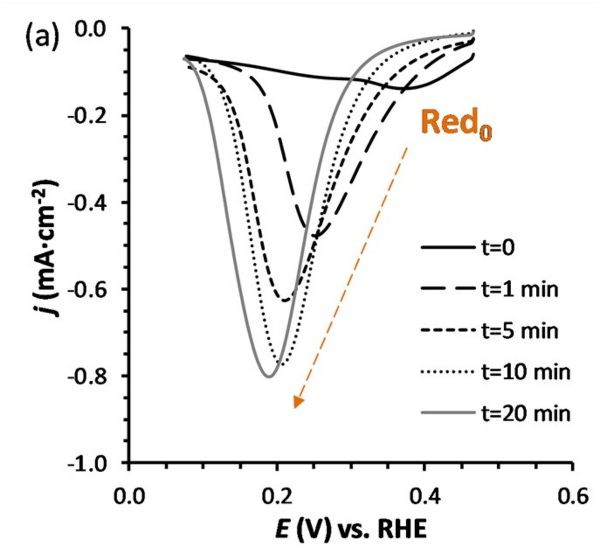


*Figure S16. (a) LSV experiment after Cu-wire electrode exposition into the electrolyte solution at time t = 0 (bold) and after 1 minute (long-dashed black), 5 min (short-dashed black), 10 min (dotted black) and 20 min (bold grey) in 0.1 M fructose and 0.12 M PBS at pH = 11.3. b) first (bold) and second (dashed) scan of the LSVs of a Cu-wire electrode right after electrochemical setup preparation (t = 0, black) and after 20 min at the OCV (grey). The LSVs were run at a 50 mV·s$^{-1}$ scan rate.*

The oxidised Cu can be reduced back to $Cu^0$ potentiometrically at pH = 11.3, for instance by applying a cathodic sweep from $E_i$ = 0.47 V to $E_{max}$ = $E_f$ = 0.07 V vs RHE ($E_i$: initial potential; $E_{max}$: maximum potential; $E_f$: final potential), as can be seen in Figure S16b. In this potential range, a reductive peak appears at ca. 0.37 V which we named as $Red_0$. As it is shown in Figure S16a, the longer the exposition time of the Cu-wire into the solution, the larger the area of the $Red_0$ peak is, and the more the peak shifts towards negative potentials. The area of this peak seems to correspond to the amount of oxidised Cu species. Although we have no analytical prove of which specific Cu species are being formed, this change on the reductive peak potential may be driven by the presence of more than one species, namely CuOH and $Cu_2O$, or even $Cu(OH)_2$ and CuO. $Red_0$ totally disappears at the second scan (Figure S3b, dashed) regardless how broad the area of the peak is, suggesting that the oxidation corresponds only to a small number of Cu species, probably via surface corrosion/passivation due to the alkalinity of the solution, as reported in the literature.[1]

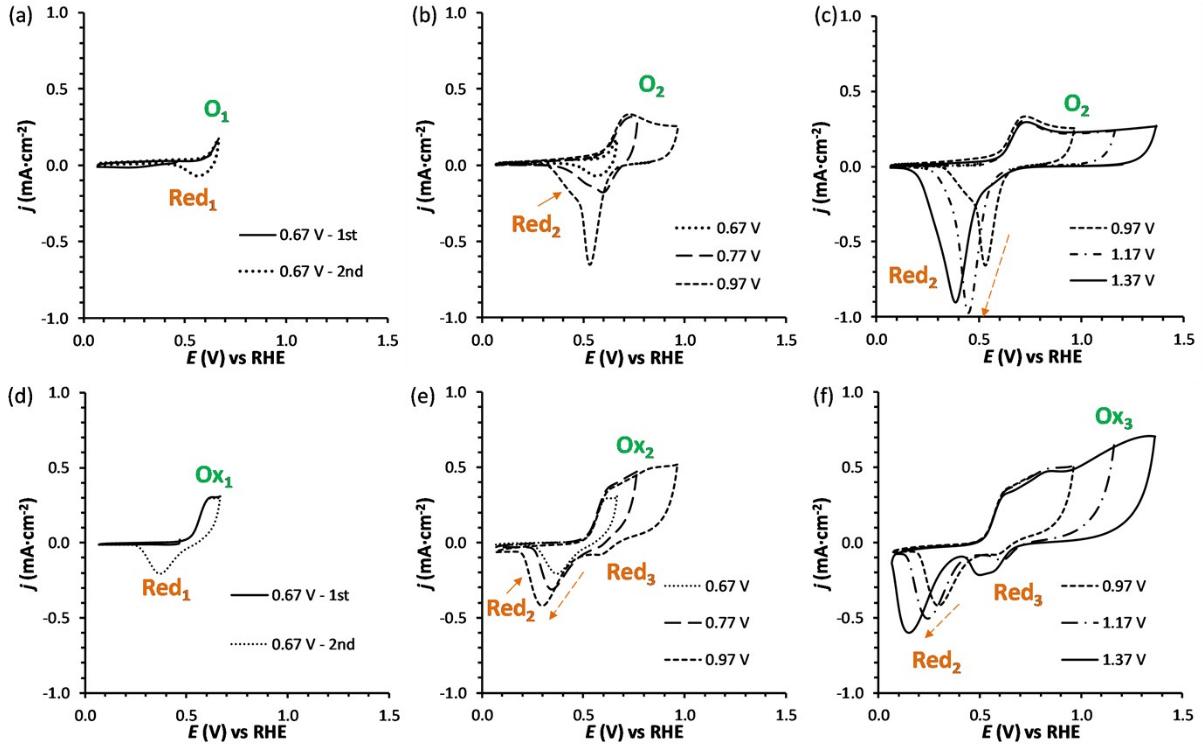


*Figure S17. Top, CVs of a Cu-wire electrode in a 0.12 M PBS solution at pH = 11.3 starting at different potentials. The electrode was electroreduced by a LSV from $E_i$ = 0.47 V to $E_f$ = 0.07 V vs RHE, previous to the plotted CVs. Bottom, same experiment in the presence of 0.1 M fructose. The CVs were run at a 50 mV·s$^{-1}$ scan rate.*

In a CV experiment within the same potential range ($E_i$ = $E_f$ = 0.47 V, $E_{max}$ = 0.07 V, Figure S17a), after reducing the passivated Cu via the just described methodology, if the $E_f$ = 0.47 V is shifted towards oxidative potentials ($E_f$ = 0.67 V; see Experimental section for further details), an increase on the current density at the anodic scan is observed ($Ox_1$), which is accompanied by the appearance of a reductive peak in the cathodic scan ($Red_1$). According to the literature, this redox couple corresponds to the 1e$^-$ transfer between Cu and $Cu_2O$ (Figure S18 and Pourbaix diagram in Figure S14 for more details).[3,21,22]

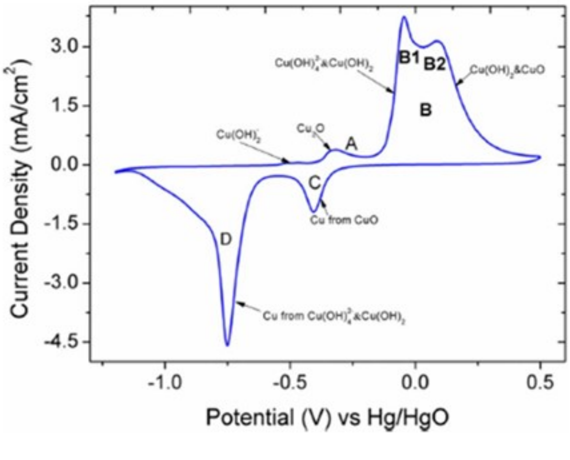


*Figure S18. CV of a bulk Cu electrode (0.5 mm dia.) in Ar-saturated 0.5 M KOH at a scan rate of 50 mV·s$^{-1}$, showing the oxidation and reduction of Cu at the different potentials and the expected species being formed at each potential. Figure reproduced with permission from reference 22.*

If the CV is run to a further oxidative potential ($E_f$ = 0.97 V; Figure S17b), we can see that $Ox_1$ does not decrease back to the capacitive current, but on the contrary keeps a constant oxidative current. When

$E_f$ is increased up to 1.37 V (Figure S17c), this oxidative current slowly increased ($Ox_2$ region). This oxidation is firstly coupled with a reduction peak $Red_2$ at ca. 0.47 vs RHE. According to the literature (see Figure S18 and Pourbaix diagram in Figure S14), the $Ox_2$/$Red_2$ pair of peaks corresponds to the further electron transfer between $Cu_2O$ and CuO.[21] However, the $Red_2$ peak position and area change when increasing $E_f$ until 1.37 V (Figure S17c), similarly to what was described in the previous paragraph and related to the Cu electrode passivation. The formation of $Cu(OH)_2$ may also take place at these potentials,[21] and thus more than one reduction peak in the cathodic scan should be expected, considering different $Cu^{II}$ species are being formed. Additionally, $Cu^{I}$ species may also coexist at this potential range, namely $Cu_2O$ and CuOH. An electrode passivation phenomenon with the formation of a double layer was also reported: the inner one made of $Cu_2O$ and the outer of $CuO/Cu(OH)_2$.[21] All these possibilities may lead to the overlapping of more than one reductive peak in the cathodic scan, thus hampering the correlation between peaks and specific redox reactions.

After the addition of 0.1 M fructose in solution (Figure S17d-f), the same set of experiments was performed in order to study the influence of the substrate to the Cu redox properties. The main outcome we observed in the CVs in the presence of fructose is the continuous increase on the oxidative current density after $Ox_1$ (0.67 V): the current does not decrease to reach the $Ox_2$ region but a new oxidation peak appears instead ($Ox_3$, see Figure S17e). Additionally, the reductive peaks $Red_1$ and $Red_2$, are shifted towards more reductive potentials and display a smaller peak area than the same peaks in the absence of fructose. Our hypothesis is that these two phenomena are related to the adsorption of fructose onto the surface of the Cu electrode. The continuous increase on the oxidative current is attributed to the oxidation of fructose species adsorbed on the surface of the Cu electrode. As it has been observed in the literature, the oxidation of glucose on Cu starts at 0.6 V vs RHE at pH $\geq$ 11, after the formation of $Cu_2O$.[23] This potential is very similar to the one we observed for fructose after $Ox_1$ Cu oxidation peak. The lower intensity of $Red_1$ and $Red_2$ peaks could also be related to the adsorption of these molecules, blocking the metallic species towards their oxidation. Finally, when reaching the more oxidative potentials, new reductive peaks appear in the cathodic scan at around 1.17 V ($Red_3$).

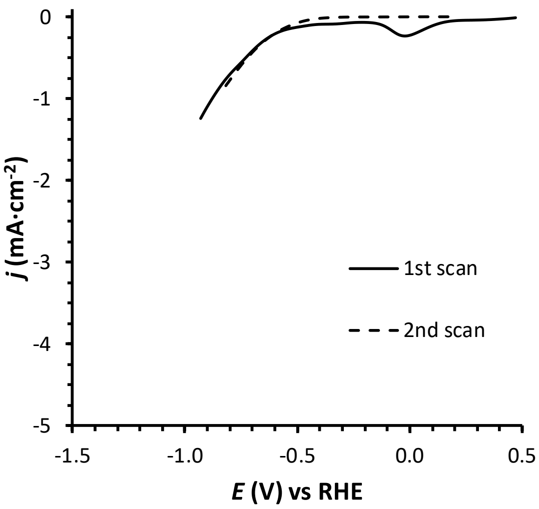


*Figure S19. First (solid) and second (dashed) LSV curves of a Cu-wire electrode in 0.1 M $Na_2SO_4$ at pH = 6.8 in the presence of 0.1 M glucose. The voltammetries were run at a 10 mV·$s^{-1}$ scan rate at room temperature (21 °C) with a Nafion membrane separator. The reduction peak observed at ca. 0 V is attributed to the reduction of oxidised Cu species (see above), and is generally observed only in the first scan.*

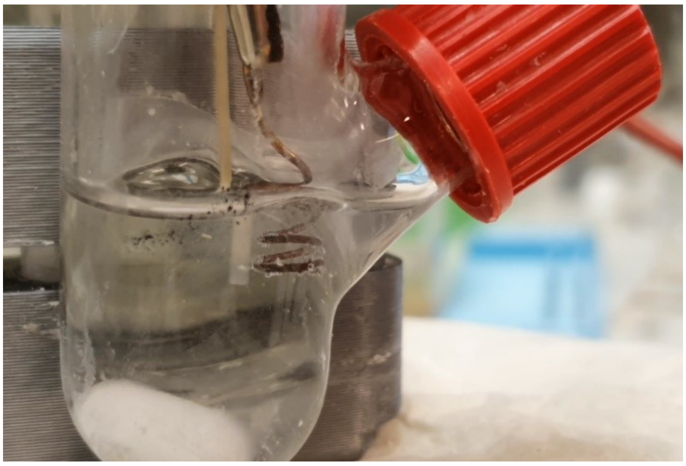

*Figure S20. Picture of the cathodic compartment with the Cu-wire, showing the formation of bubbles due to the production of $H_2$. The presence of bubbles and thus the rate of $H_2$ production increased with the increase of current density. Note that this picture was taken in a preliminary study in which a shorter coiled Cu-wire electrode was used compared to the one employed in the rest of this work (see Figure S2). The length of the coil was increased after this test to produce enough moles of product to be detected via HPLC.*

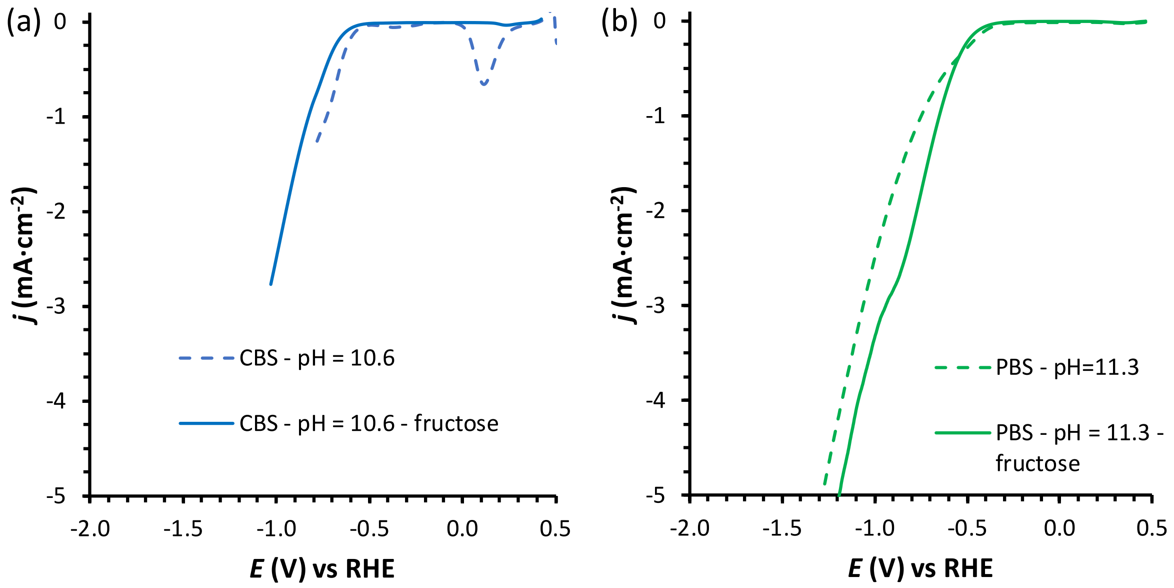


*Figure S21. LSV curves of a Cu-wire electrode in: (a) 0.5 M carbonate buffer solution (CBS) at pH = 10.6 (blue); and (b) 0.12 M PBS pH = 11.3 (green) in the absence (dashed) and presence (full) of 0.1 M fructose. The voltammetries were run at a 10 mV·s$^{-1}$ scan rate at room temperature (21 °C) with a Nafion membrane separator.*

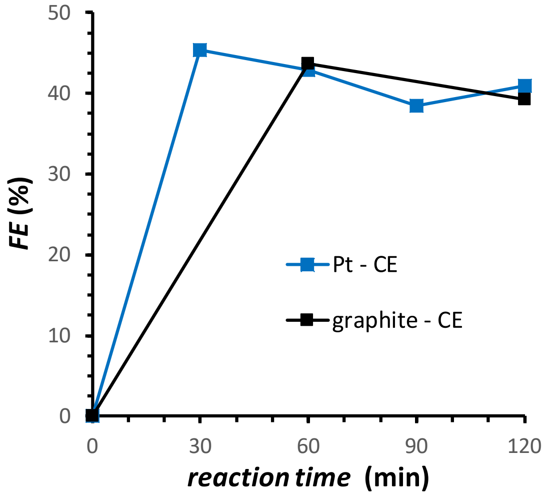


*Figure S22. Faradaic efficiency for FRR from a chronopotentiometric study on a Cu-wire electrode in a 0.12 M PBS solution at pH$_{initial}$ = 11.3 using a Pt counter electrode (blue) and a graphite counter electrode (black). Conditions: i = -20 mA (j = 5.3 mA·cm$^{-2}$), AMVN membrane separator, room temperature (21 °C).*

## References

[1] Kwon, Y.; Koper, M. T. M. *ChemSusChem* **2013**, *6* (3), 455–462.

[2] Celante, V. G.; Freitas, M. B. J. G. *J. Appl. Electrochem. 2009 402* **2009**, *40* (2), 233–239.

[3] Strehblow, H. -H; Speckmann, H. -D. *Mater. Corros.* **1984**, *35* (11), 512–519.

[4] Wang, L.; Zhang, K.; Hu, Z.; Duan, W.; Cheng, F.; Chen, J. *Nano Res.* **2013**, *7* (2), 199–208.

[5] Cudennec, Y.; Lecerf, A.; Cudennec, Y.; Lecerf, A.; State, S. **2020**, *5*, 1471–1474.

[6] Phul, R.; Kaur, C.; Farooq, U.; Ahmad, T. *Mater. Sci. Eng. Int. J.* **2018** *2* (4).

[7] He, Q.; Tian, Y.; Wu, Y.; Liu, J.; Li, G.; Deng, P.; Chen, D. *Biomol.* **2019**, *9* (5), 176.

[8] Feng, L.; Zhang, C.; Gao, G.; Cui, D. *Nanoscale Res. Lett.* **2012**, *7* (1), 1–10.

[9] Zhu, D.; Wang, L.; Yu, W.; Xie, H. *Sci. Reports* **2018**, *8* (1), 1–12.

[10] Yei, L. H. E. E.; Beden, B.; Lamy, C.; A, R. S. S.; Yei, L. H. E. E.; Beden, B.; Lamy, C.; Poitiers, D. *J. Electroanal. Chem.* **1988**, *246* (2), 349–362.

[11] Largeaud, F.; Kokoh, K. B.; Beden, B.; Lamy, C. *J. Electroanal. Chem.* **1995**, *397* (1–2), 261–269.

[12] Principles of Food Chemistry - John M. deMan, John W. Finley, W. Jeffrey Hurst, Chang Yong Lee - Google Books (accessed Jun 22, 2020).

[13] Bin Kassim, A.; Rice, C. L.; Kuhn, A. T. *J. Chem. Soc., Faraday Trans. 1* **1981**, 77 (3), 683–695.

[14] LM, H.; JS, W. *Am. J. Clin. Nutr.* **1993**, *58* (5).

[15] Carraher, J. M.; Fleitman, C. N.; Tessonnier, J.-P. *ACS Catal.* **2015**, *5* (6), 3162–3173.

[16] Cockman, M.; Kubler, D. G.; Oswald, A. S.; Wilson, L. **2006**, *8303*.

[17] Observation of the keto tautomer of d-fructose in $D_2O$ using 1H NMR spectroscopy | Elsevier Enhanced Reader(accessed Aug 20, 2021).

[18] Funcke, W.; von Sonntag, C.; Triantaphylides, C. *Carbohydr. Res.* **1979**, *75* (C), 305–309.

[19] Bin Kassim, A.; Rice, C. L. *J. Appl. Electrochem.* **1981**, 261–267.

[20] S. D. Giri ; A. Sarkar *J. Electrochem. Soc.* **2016**, *163* (3), 252

[21] Speck, F. D.; Cherevko, S. *Electrochem. commun.* **2020**, *115*, 106739.

[22] Caballero-briones, F.; Arte, J. M.; Di, I. **2009**, *111*, 1028–1036.

[23] Marioli, J. M.; Kuwana, T. *Electrochim. Acta* **1992**, *37* (7), 1187–1197.
